# Role of insulin signaling dysregulation in pulmonary vascular remodeling in rats with monocrotaline-induced pulmonary arterial hypertension

**DOI:** 10.3389/fcvm.2025.1543319

**Published:** 2025-03-24

**Authors:** Gufeng Gao, Ai Chen, Yan Yan, Mohammad Ismail Hajary Sagor, Weijun Lin, Huakan Lin, Guili Lian, Liangdi Xie, Li Luo

**Affiliations:** ^1^ Department of Geriatrics, The First Afﬁliated Hospital of Fujian Medical University, Fuzhou, Fujian, China; ^2^ Fujian Hypertension Research Institute, The First Afﬁliated Hospital of Fujian Medical University, Fuzhou, Fujian, China; ^3^ Clinical Research Center for Geriatric Hypertension Disease of Fujian Province, The First Afﬁliated Hospital of Fujian Medical University, Fuzhou, Fujian, China; ^4^ Branch of National Clinical Research Center for Aging and Medicine, The First Afﬁliated Hospital of Fujian Medical University, Fuzhou, Fujian, China; ^5^ Department of Geriatrics, National Regional Medical Center, Binhai Campus of the First Affiliated Hospital, Fujian Medical University, Fuzhou, Fujian, China

**Keywords:** pulmonary arterial hypertension, pulmonary arterial smooth muscle cells, insulin receptor substrate 1, cell proliferation, migration

## Abstract

**Background:**

Pulmonary arterial hypertension (PAH) is a severe disease marked by the remodeling of arteries due to the abnormal growth of vascular cells, including pulmonary arterial smooth muscle cells (PASMCs). The insulin receptor substrate-1 (IRS-1) plays a crucial role in the insulin signaling pathway; however, its function in PAH is still not fully understood. The objective of this research was to explore the role of the protein kinase C (PKC)/IRS-1/ERK signaling pathway in the progression of PAH and its influence on the proliferation and migration of PASMCs.

**Methods:**

To establish the PAH model, low-dose Monocrotaline (MCT) was intraperitoneally administered to male SD rats twice a week. Four weeks following the initial treatment, measurements of mean pulmonary arterial pressure (mPAP) and the right ventricular hypertrophy index (RVHI) were conducted. Additionally, calculations were performed to determine the percentage of wall area (WA%) and wall thickness (WT%). The protein levels of PKC, p-PKC, IRS-1, p-IRS-1 (Ser318), ERK, and p-ERK in lung tissues were assessed. *in vitro* experiments involved stimulating PASMCs with platelet-derived growth factor-BB (PDGF-BB) to promote proliferation and migration. The impact of the PKC inhibitor Gö 6983 and IRS-1 overexpression via adenoviral vectors (AdIRS-1) on the PKC/IRS-1/ERK signaling pathway and PASMCs behavior was analyzed through Western blotting, EdU incorporation assay, and wound healing assay.

**Results:**

In PAH rats, there was a significant rise in mPAP and RVHI (*p* < 0.05), accompanied by notable pulmonary vascular remodeling. Analysis of lung tissues revealed enhanced levels of p-PKC, p-IRS-1(Ser318), and p-ERK, whereas the expression of total IRS-1 decreased significantly (*p* < 0.05). In PASMCs stimulated with PDGF-BB, a similar trend of increased p-PKC, p-IRS-1(Ser318), and p-ERK levels was observed, along with a decrease in IRS-1 expression. The administration of Gö 6983 or the overexpression of IRS-1 effectively inhibited the activation of the PKC/IRS-1/ERK signaling pathway, leading to reduced proliferation and migration of PASMCs compared to stimulation with PDGF-BB alone (*p* < 0.05).

**Conclusions:**

The PKC/IRS-1/ERK signaling pathway is implicated in the abnormal proliferation and migration of PASMCs, contributing to pulmonary vascular remodeling in PAH. Targeting this pathway through PKC inhibition or IRS-1 stabilization may offer novel therapeutic strategies for PAH management.

## 1 Introduction

Pulmonary arterial hypertension (PAH) is a severe disease characterized by arterial remodeling, which leads to elevated vascular resistance, increased right ventricular afterload, and the development of heart failure (1). Despite advancements in treatment, current therapies primarily slow disease progression rather than reversing the underlying pathology (2–4). The excessive proliferation and migration of pulmonary arterial smooth muscle cells (PASMCs) are key contributors to the vascular remodeling observed in PAH (5). Therefore, understanding the mechanisms driving PASMCs proliferation and migration is essential for developing effective therapies.

Emerging evidence implicates metabolic dysregulation, including insulin resistance (IR), in PAH pathogenesis (6–8). Insulin receptor substrate (IRS) proteins are central mediators of insulin signaling and play vital roles in regulating cellular growth and metabolism (9). Four members (IRS-1, IRS-2, IRS-3, and IRS-4) of the IRS family have been identified, which differ in their tissue distribution, subcellular localization, binding to the insulin receptor (INSR), and interactions with SH2 domain-containing proteins (9). IRS-2 has been associated with PAH in previous studies, showing reduced expression in the pulmonary vasculature (10). However, IRS-1, the most extensively studied IRS family member, is uniquely involved in mitogenic pathways and remains underexplored in PAH. Understanding the role of IRS-1 may uncover novel insights into the mechanisms driving PASMCs dysfunction.

After binding to the INSR, insulin mainly acts via two pathways: IRS-1/PI3K/AKT and IRS-1/ERK. The intracellular IRS-1/PI3K/AKT signaling pathway mainly mediates the various metabolic effects of insulin on cells. The IRS-1/ERK pathway, a key arm of insulin signaling, primarily regulates cell growth, proliferation, and migration (11–13). Under IR conditions, IRS-1 undergoes serine phosphorylation, leading to dysregulated signaling (14), reduced IRS-1 protein level, and enhanced ERK activation (15). Previous studies have shown that insulin stimulated many kinases [including protein kinase C (PKC), AKT, SIK2, and mTOR], which have mediated increased S/T phosphorylation of IRS-1 with negative effects on insulin sensitivity (16). PKC, a serine/threonine kinase, has been shown to phosphorylate IRS-1, contributing to insulin signaling disruption (16). Elevated PKC activity has been observed in PAH models and vascular remodeling (17), suggesting a potential upstream regulator of IRS-1 dysfunction.

In this study, we investigated the role of the PKC/IRS-1/ERK signaling pathway in monocrotaline (MCT)-induced PAH rats and platelet-derived growth factor-BB (PDGF-BB)-stimulated PASMCs. Our findings provide new insights into the molecular mechanisms underlying PAH and suggest potential therapeutic targets for intervention.

## 2 Materials and methods

### 2.1 Animals and the PAH model

The Laboratory Animal Welfare and Ethics Committee at Fujian Medical University granted approval for this study (Approval No. FJMU IACUC 2021-0387). Eight-week-old male Sprague-Dawley rats, each weighing between 200 and 230 grams, were sourced from Shanghai Slack Laboratory Animal Co., Ltd. [license number: SCXK (Shanghai, China) 2017-0005]. These rats were housed in the Animal Center of Fujian Medical University, where they enjoyed unrestricted access to food and water, and were kept at a temperature of 22 ± 2°C with a humidity level of 55% ± 5%. A total of sixteen rats were randomly assigned to either the control or PAH group. The PAH group received two intraperitoneal injections of 20 mg/kg of MCT (MCE, HY-N0750, USA) administered one week apart, following a protocol previously outlined by our research team (18), while the control group was given the same volume of normal saline via the same method. After four weeks post the initial MCT injection, all rats were euthanized for the assessment of mean pulmonary arterial pressure (mPAP), right ventricular hypertrophy index (RVHI), and lung histopathological examinations.

### 2.2 Hemodynamic measurements

Following the administration of an intraperitoneal injection of sodium pentobarbital at a dosage of 50 mg/kg for anesthesia, mPAP was assessed through right heart catheterization, as previously documented (18). The resulting waveform data were captured and analyzed using a PowerLab system from ADinstruments Pty Ltd in Australia. Once the hemodynamic evaluations were completed, the rats were euthanized to measure the weights of the right ventricle, left ventricle, ventricular septum, and to calculate the RVHI using the formula RVHI = RV/(LV + S). Lung tissues were then collected for subsequent histological examination.

### 2.3 Histological analysis

Hematoxylin and eosin (HE) staining was employed to examine structural alterations in the pulmonary arterioles of lung tissues. From each tissue slice, five arterioles approximately 100 µm in diameter were randomly selected, situated around 2 mm from the lung hilum. These samples were then analyzed using Image Pro Plus 6.0 software. We measured wall thickness (WT), external diameter (ED), lumen area (LA), and total area (TA) to determine the percentage of wall area (WA%) using the formula WA% = [(TA − LA)/TA] × 100%, and the percentage of wall thickness (WT%) calculated as WT% = (2 × WT/ED) × 100%. The mean values obtained served as the morphometric index for the pulmonary vasculature in rats.

### 2.4 Immunohistochemical staining

Paraffin-embedded sections were first deparaffinized to let reagents access tissue, then rehydrated. Next, they were incubated with 3% BSA for 30 min at room temperature to block non-specific sites. After that, IRS-1 antibody (1:500, Proteintech, USA) and p-IRS-1 (1:300, Immunoway, USA) were added and incubated overnight at 4°C. Sections were washed three times with PBS to remove unbound primary antibodies. A secondary antibody was added and incubated for 1 h at room temperature, followed by another three times PBS wash. Finally, DAB color-developing solution was added. The stained sections were observed under a Nikon 80i light microscope to visualize and quantify target antigens by brown staining.

### 2.5 Isolation and culture of PASMCs

Following the anesthetization and cervical dislocation of the rats, the isolated lung lobes were promptly placed into a Petri dish filled with PBS and swiftly moved to an ultra-clean workbench. The pulmonary arteries were then meticulously isolated and sliced into smaller segments based on established protocols (19, 20). To identify PASMCs, staining with α-SMA (1:200, Abbkine Scientific, China) was conducted. For the cultivation of PASMCs, Dulbecco's modified Eagle's medium/F12 (DMEM/F12, HyClone, USA) supplemented with 10% fetal bovine serum (FBS) (Gibco, Australia) was utilized. Only cells from passages two to four were employed for the subsequent experiments.

### 2.6 PASMCs treatment

To promote the growth of PASMCs, PDGF-BB (Peprotech, 100-14B-10, USA) was applied at a concentration of 20 ng/ml, following established protocols (19, 21, 22). The PASMCs were initially grown in DMEM/F12 with 10% FBS until they achieved a confluence of 80%–90%. Subsequently, the growth medium was switched to DMEM/F12 devoid of FBS, effectively starving the cells for a period of 24 h. After this period, the cells were treated with 10 μM Gö 6983 (23) for 30 min prior to being stimulated with PDGF-BB (20 ng/ml) for 48 h before collection.

### 2.7 Vector construction and cell infection

IRS-1 primers were designed according to the CDS sequence of IRS-1 (NM_012969.2) in the NCBI database and the restriction site on the plasmid vector GV230 (CMV-MCS-EGFP-SV40-Neomycin) (Shanghai Genechem Co., Ltd.). Gene amplification was performed by PCR, which was packaged and purified using a multifunctional DNA purification and recovery kit to obtain the target gene (Shanghai Genechem Co., Ltd.). The IRS-1 overexpression via adenoviral vectors (AdIRS-1) was constructed by Shanghai Hanbio Biotechnology Co., Ltd. The AdNull control was purchased from Shanghai Hanbio Biotechnology Co., Ltd. For the overexpression studies, PASMCs were transfected with AdIRS-1. To determine the optimal transfection conditions for AdIRS-1 in PASMCs, we infected PASMCs with different multiplicity of infection (MOI) values (0, 1, 5, 10, 50, 100, and 200). After 48 h of infection, western blotting and inverted fluorescence microscopy were performed to confirm the expression of enhanced green fluorescent protein (EGFP) in PASMCs.

### 2.8 Western blotting

Western blotting was performed as described in previous studies (24). After the treatment, proteins from lung tissues and PASMCs were extracted using lysis buffer. Protein concentrations in the lysates were assessed using a BCA protein assay kit (Beyotime, China). The protein samples were then subjected to separation via 8% or 10% sodium dodecyl sulfate-polyacrylamide gel electrophoresis, followed by transfer to PVDF membranes. The membranes were subsequently blocked with 5% nonfat milk for 1 h. Following this, the blots were incubated overnight at 4°C with monoclonal antibodies: anti-PKCδ (1:500; Santa Cruz, USA), anti-phospho-PKCδ (1:500; Santa Cruz, USA), anti-IRS-1 (1:500; Merck Millipore, Germany), anti-phospho-IRS-1 (ser318) (1:500; Cell Signaling Technology, USA), anti-ERK (1:1,000; Cell Signaling Technology, USA), and anti-phospho-ERK (1:1,000; Cell Signaling Technology, USA). The PVDF membranes underwent three washes with TBST, each lasting 10 min, and were then incubated with the appropriate secondary antibody for 1 h. The blots were determined by the ECL detection system (Beyotime, China) before being further analyzed using ImageJ software (National Institutes of Health, Bethesda, MD, USA) (25).

### 2.9 Cell proliferation assay

An EdU incorporation assay (Beyotime, China) was conducted to evaluate the proliferation rate of PASMCs following a previously established protocol (20). In this assay, red fluorescence (indicative of EdU-positive cells) signifies proliferating cells, while blue fluorescence (attributed to Hoechst 33,342-stained cells) denotes the total cell population. Consequently, the proliferation rate of PASMCs was determined by calculating the proportion of proliferating cells relative to the total cell count.

### 2.10 Wound healing assay

A wound healing assay was performed to determine the migration rate of PASMCs. When the PASMCs reached 90% confluence in the six-well plates, a 200 μm pipette tip was used to scratch a straight line at the bottom of each well. Migration images were captured at 0 h and 48 h. Then, the cell migration rate was calculated as [(area of initial scratch–area of current scratch)/area of initial scratch] × 100%.

### 2.11 Statistical methods

GraphPad Prism 8 software was used to process the data and draw statistical graphs. Measurement data are expressed as mean ± standard error (mean ± SEM), and a *t*-test was used to compare the means between the two groups. One-way ANOVA was used to compare the means among multiple groups, followed by the least significant difference (LSD) *t*-test for pairwise comparisons. *P* < 0.05 indicated that the difference was statistically significant.

## 3 Results

### 3.1 Hemodynamic measurements and morphometric analysis of pulmonary arterioles in rats with PAH

**Figure 1:**
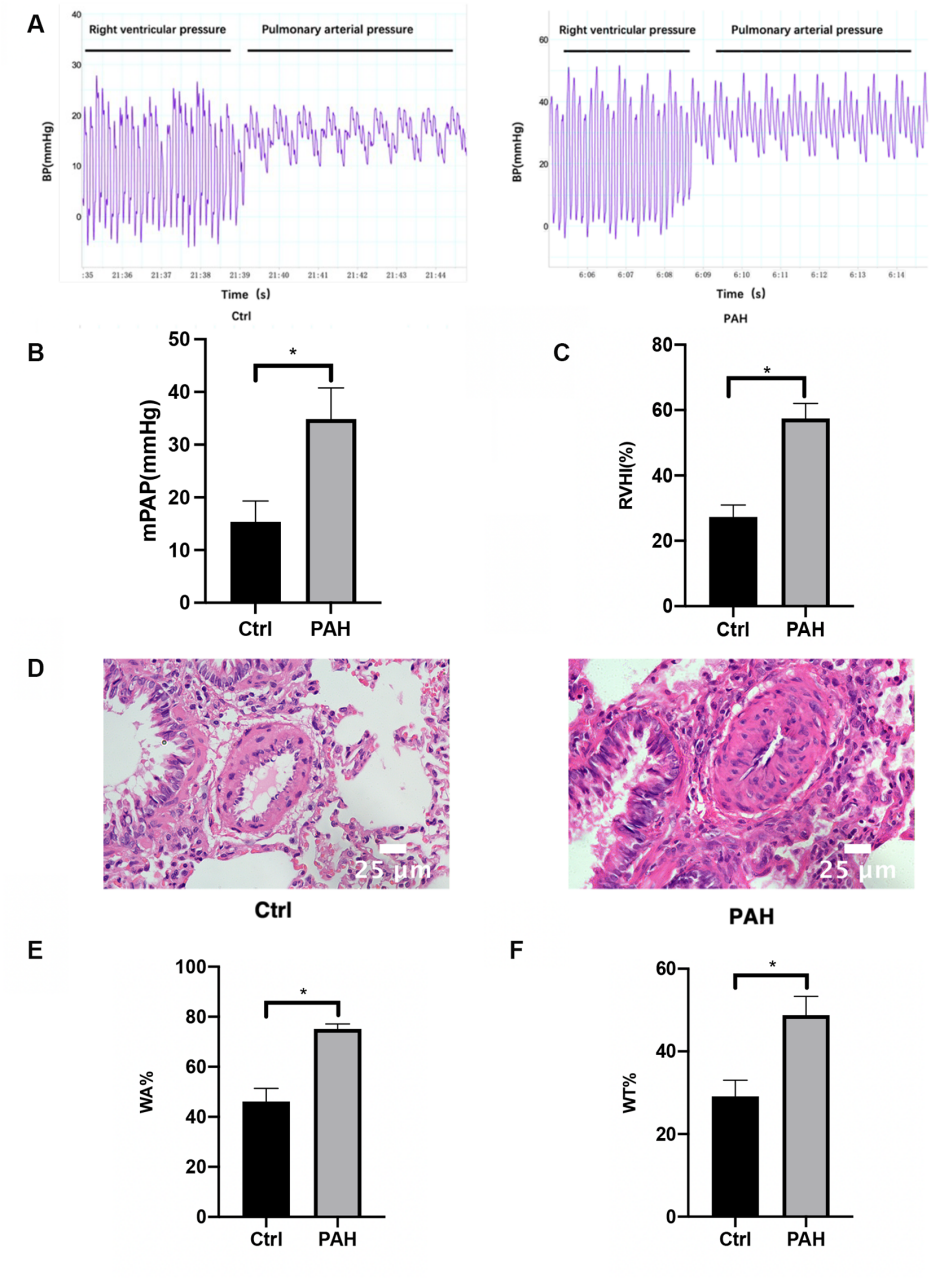
Hemodynamic measurements and morphometric analysis of pulmonary arterioles. Four weeks after the first MCT administration, right heart catheterization was used to measure the pulmonary artery pressure in rats. **(A)** The pressure waveforms were observed when the catheter was inserted into the pulmonary artery through the right ventricle. **(B)** Comparison of mPAP between the control group and the PAH group. **(C)** Changes in RVHI in PAH rats four weeks after the first MCT administration. Four weeks after the first MCT administration, the heart was removed, and the weight ratio of RV and LV + S was used as the level of RVHI and compared between the control and PAH groups. **(D)** HE staining pictures of lung tissues show the structural changes of the small arteries in the lungs of the control group and MCT-induced PAH rats. Scale bar = 25 μm. **(E,F)** Statistical results of WT% and WA% of pulmonary arterioles (diameter <100 μm) in the control group and MCT-induced PAH group 4 weeks after the first MCT administration, WT%: the percentage of vascular wall thickness; WA%: the percentage of vascular wall area. Data are expressed as mean ± standard deviation (mean ± SD), *n* = 8. *P < 0.05 vs. Ctrl. Ctrl, control group; PAH, MCT-induced pulmonary hypertension group.

As illustrated in Figure 1A, right-heart catheterization enabled the capture of PAP waveforms in rats. Furthermore, the mPAP levels in the PAH group were markedly elevated compared to the control group, as shown in Figure 1B. The RVHI for the PAH group also exhibited a statistically significant increase over the control group (Figure 1C). Subsequently, HE staining was conducted to examine the morphological alterations in lung tissues across both the control and PAH groups (Figure 1D). In the control group, the intima of the pulmonary artery appeared smooth and intact, showing no signs of inflammatory cell infiltration or necrosis. In contrast, the pulmonary artery lumen in the PAH group was considerably narrower, with a significant thickening of the intima and extensive infiltration of inflammatory cells. Moreover, WA% and WT% were critical parameters for assessing the degree of remodeling in the pulmonary vasculature. The WA% and WT% associated with the MCT-induced PAH group were significantly greater than those observed in the control group (Figures 1E,F). In conclusion, the establishment of the MCT-induced PAH rat model has been successfully achieved.

### 3.2 Activity of the PKC/IRS-1/ERK signaling pathway is increased in the lung tissues of PAH rats

**Figure 2:**
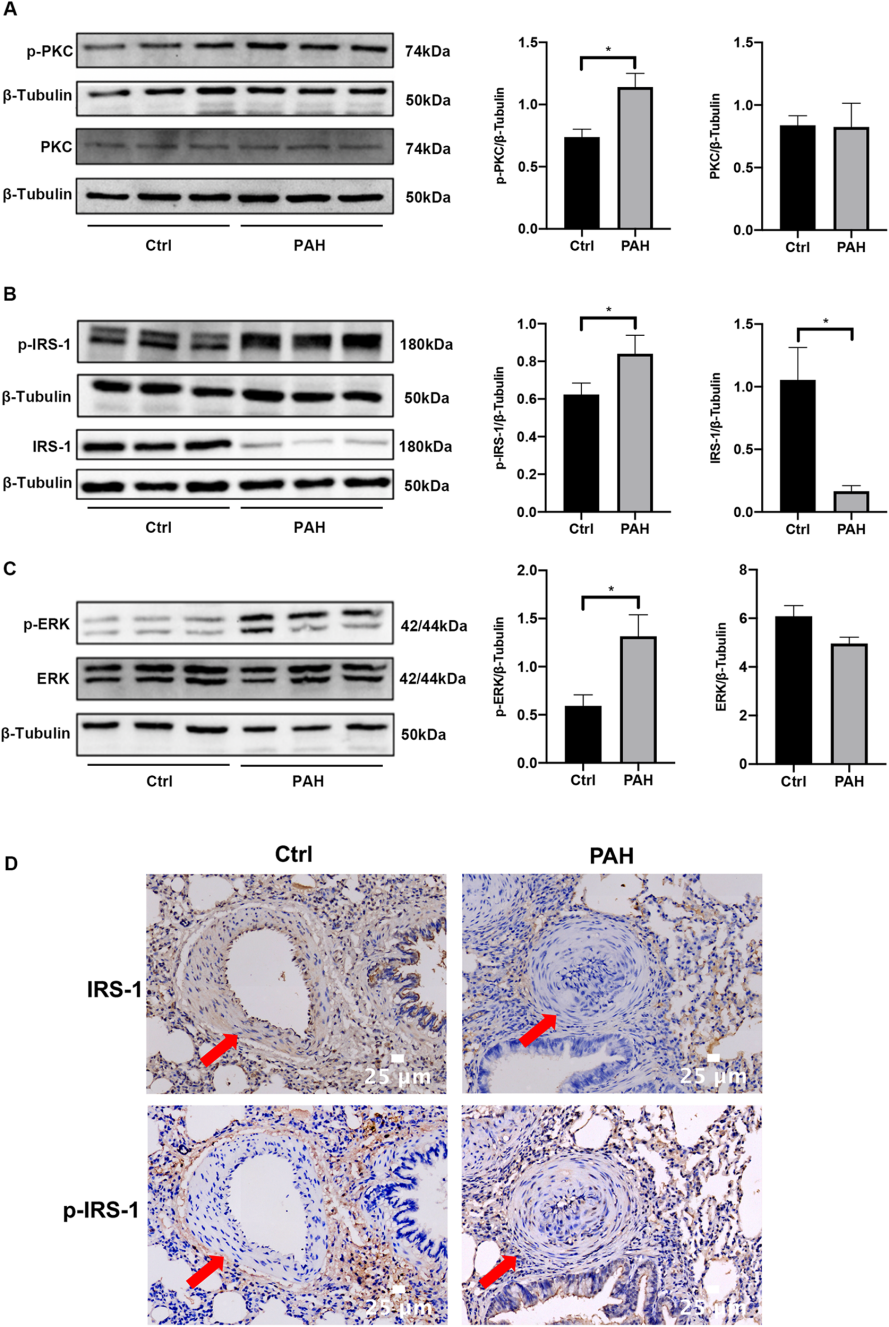
The PKC/IRS-1/ERK signaling pathway activity was increased in lung tissues. **(A)** Western blotting was used to detect the expression changes of PKC and p-PKC in the lung tissues of the control group and PAH rats four weeks after the first intraperitoneal injection of MCT. **(B)** The expressions of IRS-1 and p-IRS-1 were detected in the lung tissues of control and PAH rats. **(C)** Western blotting was performed to understand the expression changes of ERK and p-ERK in the lung tissues of the control and PAH rats four weeks after the first intraperitoneal injection of MCT. **(D)** Immunohistochemistry was employed to detect the expression changes of IRS-1 and p-IRS-1 in the lung tissues of the control and PAH rats. Data are expressed as mean ± standard deviation (mean ± SD), *n* = 8. *P < 0.05. Ctrl, control group; PAH, MCT-induced pulmonary hypertension group.

Four weeks after the initial MCT injection, we utilized western blotting to assess changes in the expression levels and phosphorylation statuses of PKC, IRS-1, and ERK within the pulmonary tissues of rats (Figures 2A–C). Meanwhile, immunohistochemistry was employed to detect the expression levels of IRS-1 and phosphorylated IRS-1 in the lung tissues (Figure 2D). In comparison to the control group, there were no significant differences in PKC and ERK expression in the lung tissues of rats with PAH; however, a notable decrease in IRS-1 protein levels was observed in the PAH group. Additionally, the phosphorylation levels of PKC, IRS-1, and ERK in the lung tissues were found to be elevated when compared to those in the control group, indicating that the PKC/IRS-1/ERK signaling pathway's activity was increased in the lung tissues of MCT-treated rats.

### 3.3 Cell identification

**Figure 3:**
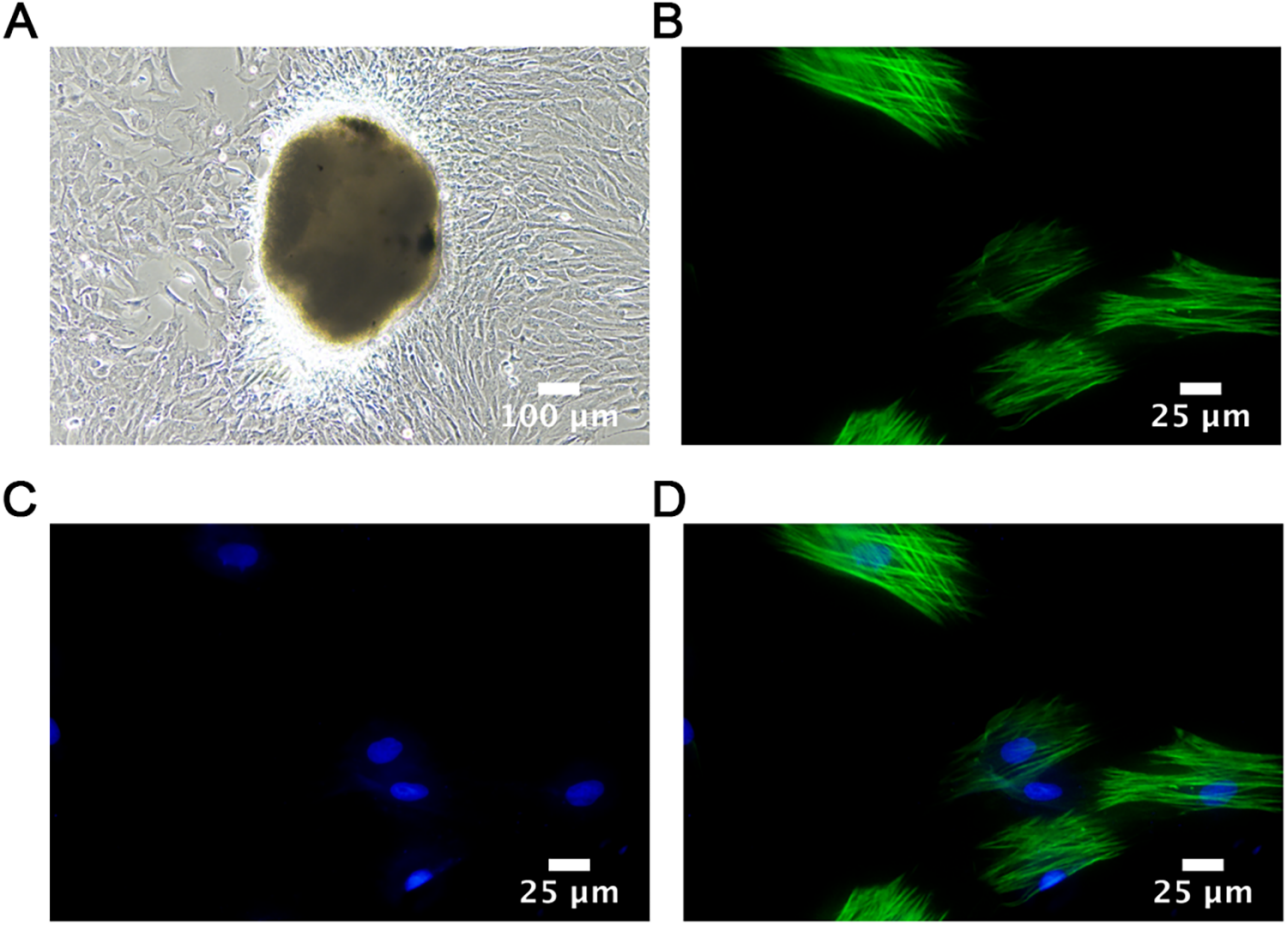
Cell identification. **(A)** Adherent cultured rat PASMCs (1st week); Scale bar = 100 μm. **(B–D)** Immunofluorescence images of PASMCs identified by α-SMA staining taken by confocal immune microscopy. Scale bar = 25 μm.

After isolation and mincing, rat pulmonary artery smooth muscle tissue was cultured. Observations after seven days showed that the primary rat PASMCs were arranged radially around the pieces of tissue, most of which were spindle-like with flat shapes and clear edges. For cell identification, immunofluorescence staining was performed. As shown in Figure 3, the nucleus was visible and green myofilaments were observed in the cytoplasm, confirming that the cultured cells were PASMCs.

### 3.4 Activity of the PKC/IRS-1/ERK signaling pathway in PASMCs was elevated after PDGF-BB stimulation

**Figure 4:**
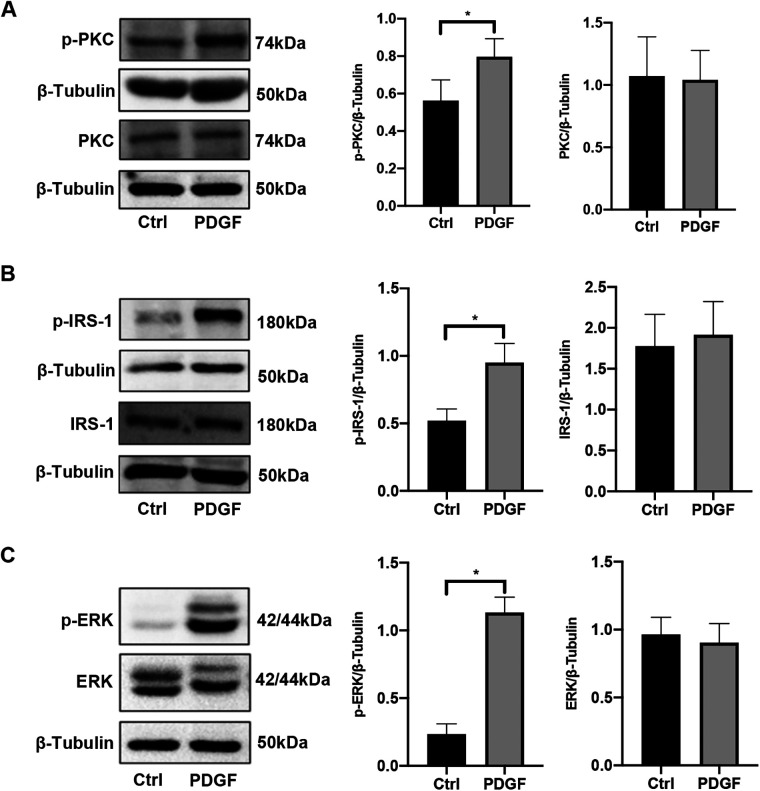
The activity of the PKC/IRS-1/ERK signaling pathway in PASMCs was elevated after PDGF-BB stimulation. **(A)** Western blotting was used to detect the changes of PKC and p-PKC in PASMCs after PDGF-BB induction for 15 min. **(B)** The levels of IRS-1 and p-IRS-1 were observed in PASMCs after PDGF-BB induction for 15 min. **(C)** The expression changes of ERK and p-ERK were ensured using western blotting. Data are expressed as mean ± standard deviation (mean ± SD), *n* = 5. *P < 0.05. Ctrl, control group; PDGF, PASMCs induced by PDGF-BB for 15 min.

After PDGF-BB treatment for 15 min, the PASMCs were collected for western blotting. As shown in Figure 4, the protein levels of PKC, IRS-1, and ERK remained unchanged. However, the expression levels of p-PKC, p-IRS-1 (ser318), and p-ERK were significantly elevated compared with those in the control group. This indicates that the PKC/IRS-1/ERK pathway was activated after 15 min of PDGF-BB intervention, which is consistent with the results we observed at the animal level.

### 3.5 PKC inhibitor Gö 6983 inhibited the activity of the PKC/IRS-1/ERK signaling pathway in PDGF-BB-treated PASMCs

**Figure 5:**
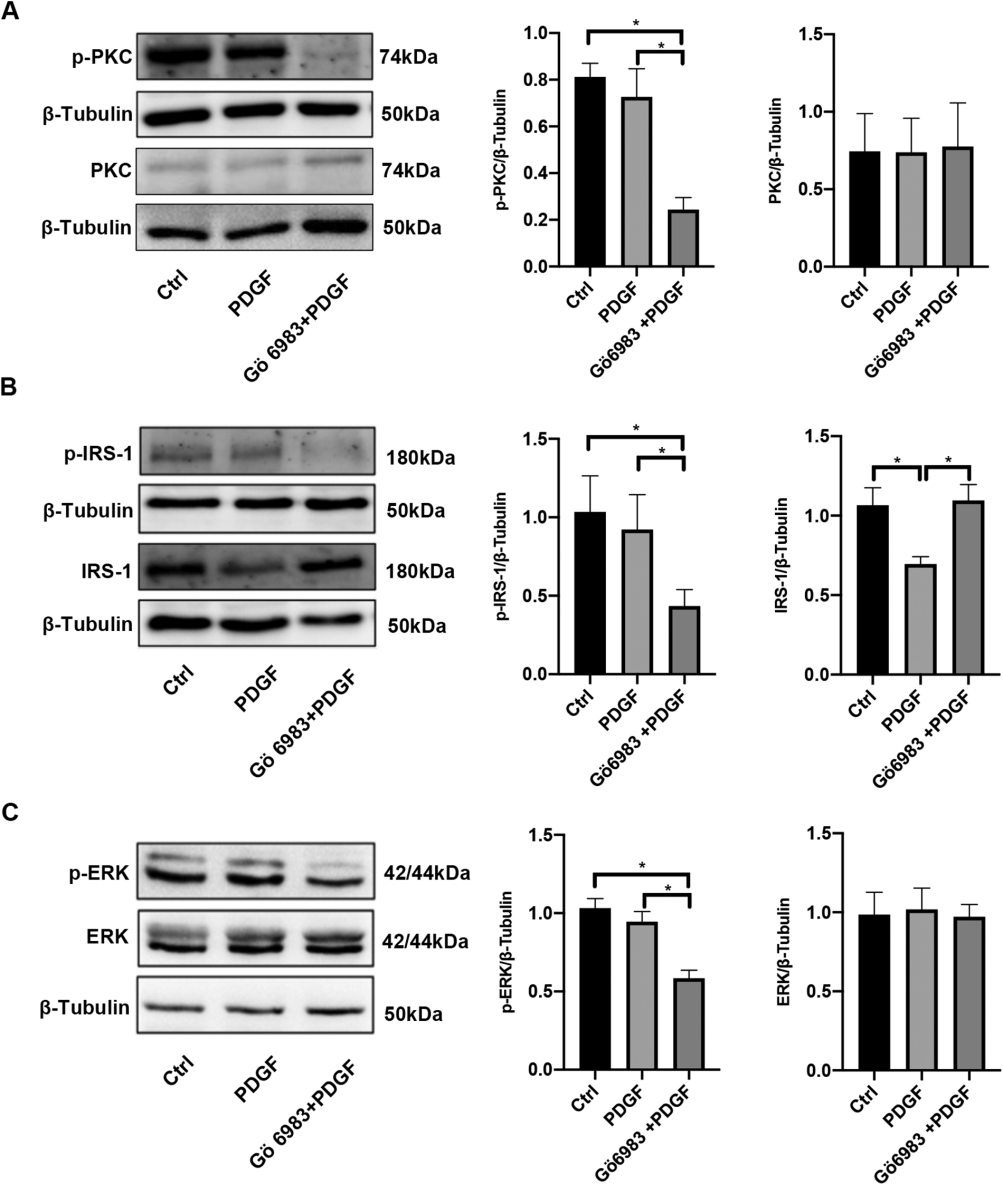
PKC inhibitor Gö 6983 inhibited the activity of the PKC/IRS-1/ERK signaling pathway in PDGF-BB-induced PASMCs. PASMCs were serum-free starved for 24 h, pretreated with PKC inhibitor Gö 6983 (10 μM) for 30 min, then induced by PDGF-BB (20 ng/ml) for 48 h. **(A)** Western blotting was used to detect the changes of PKC and p-PKC in PASMCs after the induction of PDGF-BB and Gö 6983. **(B)** The levels of IRS-1 and p-IRS-1 were observed in PASMCs after the induction of PDGF-BB and Gö 6983. **(C)** The expression changes of ERK and p-ERK were ensured using western blotting after the induction of PDGF-BB and Gö 6983. Data are expressed as mean ± standard deviation (mean ± SD), *n* = 5. *P < 0.05. Ctrl, control group; PDGF, PASMCs induced by PDGF-BB for 48 h; Gö 6983 + PDGF, PASMCs pretreated Gö 6983 for 30 min, then induced by PDGF-BB for 48 h.

Following 48 h of PDGF-BB stimulation, the levels of PKC, p-PKC, p-IRS-1 (ser318), ERK, and p-ERK remained unchanged, although the IRS-1 protein underwent degradation during this period. In contrast, treatment with Gö 6983 resulted in reduced levels of p-PKC, p-IRS-1 (ser318), and p-ERK, while the expressions of PKC and ERK showed no significant alterations. Importantly, the administration of Gö 6983 counteracted the decreasing trend of IRS-1 expression in PASMCs stimulated with PDGF-BB for 48 h (Figure 5). Therefore, after 48 h, the phosphorylation levels of the PKC/IRS-1/ERK pathway in the PDGF-intervened cells showed no difference compared with those in the control group. However, at this point, the inhibitory effect of Gö 6983 on the PKC/IRS-1/ERK pathway could still be observed, indicating that even after 48 h, the inhibitory effect of Gö 6983 on the PKC/IRS-1/ERK pathway remained remarkable.

### 3.6 PKC inhibitor Gö 6983 inhibited the proliferation and migration of PASMCs induced by PDGF-BB

**Figure 6:**
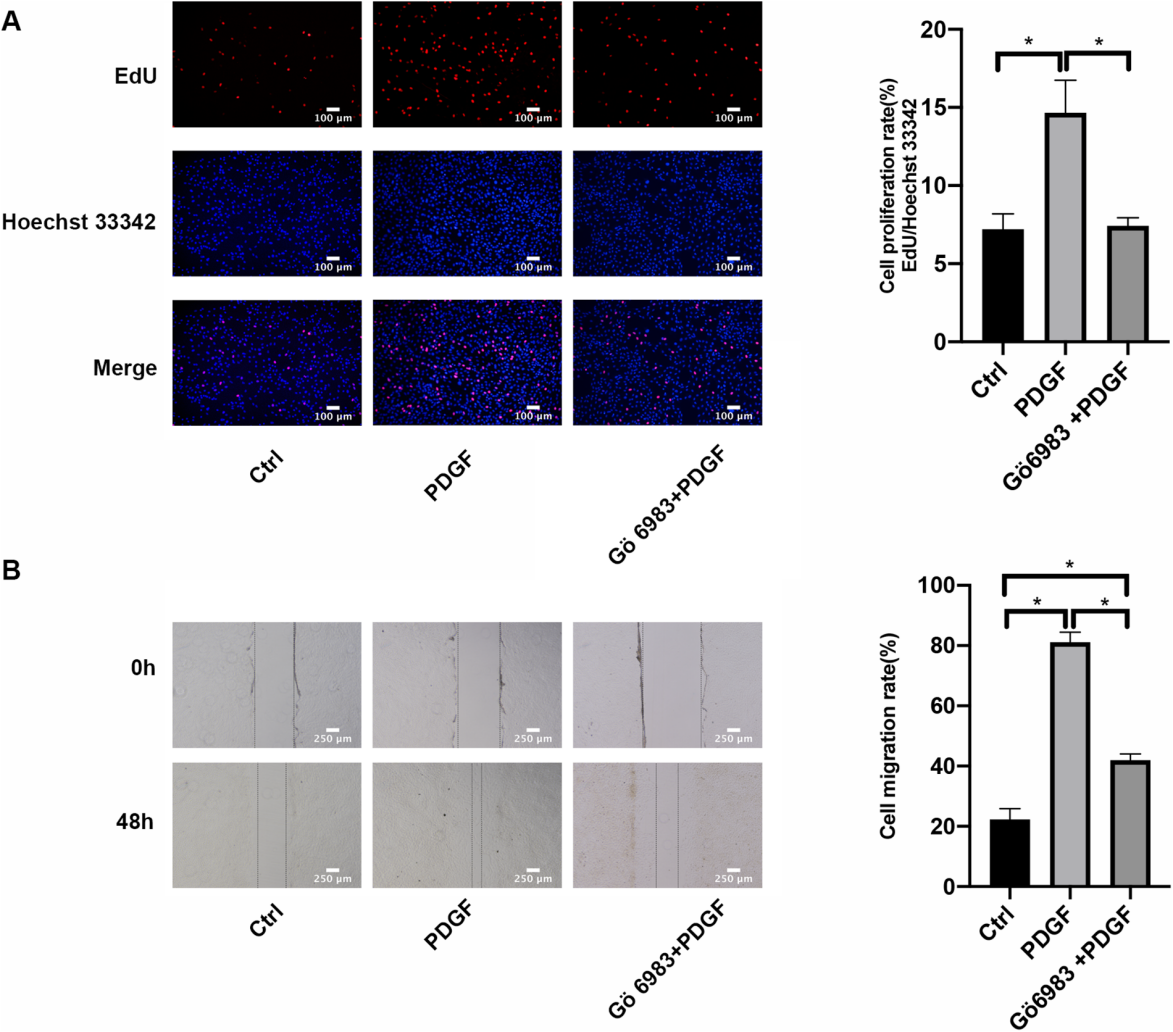
PKC inhibitor Gö 6983 inhibited the proliferation and migration rates in PDGF-BB-induced PASMCs. **(A)** The effect of PKC inhibitor Gö 6983 on the proliferation of PASMCs induced by PDGF-BB by EdU incorporation assay. PASMCs were serum-free starved for 24 h, pretreated with Gö 6983 (10 μM) for 30 min, and then added 20 ng/ml PDGF-BB to intervene in cells for 48 h. The level of cell proliferation was detected by EdU incorporation assay. Scale bar = 100 μm. **(B)** The effect of PKC inhibitor Gö 6983 on the migration level of PASMCs induced by PDGF-BB by the wound healing assay. After starvation in serum-free medium for 24 h, we scratched the bottom of the 6-well plate with a 1 ml sterile pipette tip, pretreated the cells with Gö 6983 (10 μM) for 30 min, and then added PDGF-BB (20 ng/ml) to induce cells for 48 h. Images were taken at 0 h and 48 h. Scale bar = 250 μm. Data are expressed as mean ± standard deviation (mean ± SD), *n* = 5. *P < 0.05. Ctrl, control group; PDGF, 20 ng/ml PDGF-BB-induced PASMCs group; Gö 6983 + PDGF, PASMCs pretreated with Gö 6983 for 30 min, then induced by PDGF-BB for 48 h.

In comparison to the control group, after 48 h of PDGF-BB treatment, there was a significant increase in the proliferation rates of PASMCs as determined by EdU incorporation assay; however, the intervention with Gö 6983 partially mitigated this increase (Figure 6A). The findings from the wound healing assay indicated that the migration rate of PASMCs stimulated by PDGF-BB was considerably greater than that of the control group at the 48-hour mark. Although the effect was somewhat reduced by the Gö 6983 intervention, the migration rate in this group remained higher than that of the control at the same time point (Figure 6B). In summary, Gö 6983 effectively decreased both the proliferation and migration rates of PASMCs that were activated by PDGF-BB.

### 3.7 Overexpressed IRS-1 inhibited the activity of the ERK signaling pathway in PASMCs

**Figure 7:**
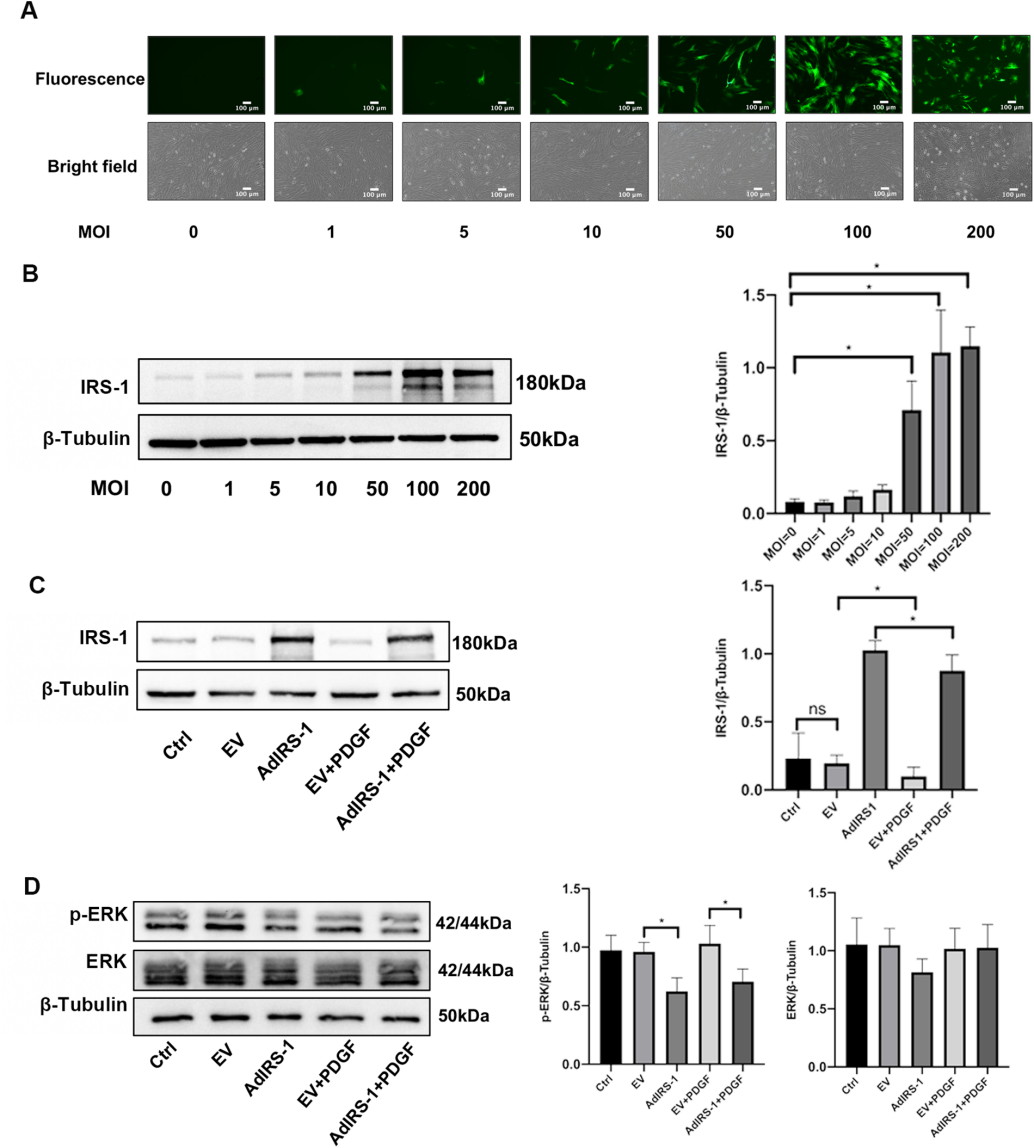
Overexpressed IRS-1 inhibited the activity of ERK signaling pathway in PASMCs. **(A)** Fluorescence images and brightfield images of PASMCs infected with AdIRS-1 with different MOI values (0, 1, 5, 10, 50, 100, 200) observed by fluorescence microscope. Scale bar = 100 μm. **(B)** The expression level of IRS-1 in PASMCs infected with AdIRS-1 with different MOI values (0, 1, 5, 10, 50, 100, 200) detected by western blot. MOI: multiplicity of infection; **(C)** The expression level of IRS-1 in PDGF-BB-induced PASMCs infected with AdIRS-1 (MOI value = 100). **(D)** The expression level of ERK and p-ERK in PDGF-BB-induced PASMCs infected with AdIRS-1 (MOI value = 100). Data are expressed as mean ± standard deviation (mean ± SD), *n* = 5. *P < 0.05. Ctrl, control group; PDGF, 20 ng/mg PDGF-BB-induced PASMCs group; EV, empty vector; AdIRS-1, adenovirus expressing EGFP and IRS-1.

The expression of IRS-1 was found to be reduced in PDGF-BB-PASMCs, leading to the use of AdIRS-1 for its overexpression in these cells for subsequent studies. To identify the most effective multiplicity of infection (MOI), different MOI values (0, 1, 5, 10, 50, and 100) of AdIRS-1 were applied to PASMCs, which were then incubated for 72 h. Data presented in Figure 7A indicated that the effectiveness of AdIRS-1 infection improved as the MOI values increased (ranging from 0 to 100); however, cell mortality was observed at an MOI of 200. Additionally, western blot analysis revealed a significant increase in IRS-1 levels in PASMCs at an MOI of 100, as illustrated in Figure 7B. Therefore, an MOI of 100 was chosen for the following experiments. To assess how overexpression of IRS-1 influences the ERK signaling pathway in PDGF-BB-PASMCs, the levels of IRS-1, phosphorylated ERK (p-ERK), and total ERK were analyzed. The infection efficiency of IRS-1 at an MOI of 100 was verified in PDGF-BB-PASMCs (Figure 7C). Additionally, it was found that overexpression of IRS-1 significantly reduced the levels of p-ERK while not affecting ERK levels (Figure 7D). In summary, the overexpression of IRS-1 diminished the activity of the ERK signaling pathway in PASMCs.

### 3.8 Overexpressed IRS-1 inhibited the proliferation and migration rate in PASMCs

**Figure 8:**
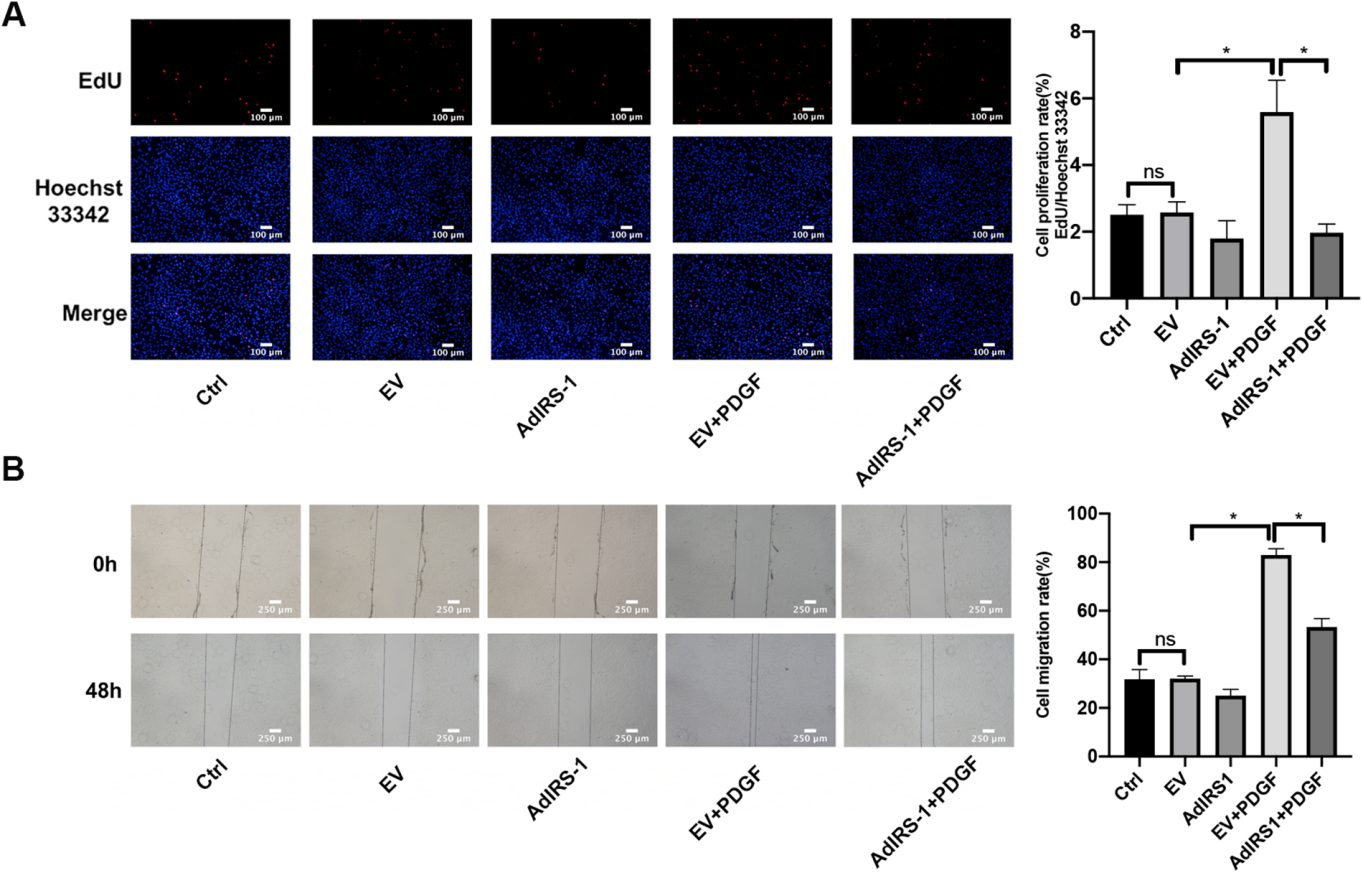
Overexpression of IRS-1 inhibited the proliferation and migration rate in PASMCs. **(A)** The effect of AdIRS-1 on the proliferation of PASMCs induced by PDGF-BB by EdU assay. After being infected with AdIRS-1 (MOI = 100) for 48 h, PASMCs were then serum-free starved for 24 h before being treated with 20 ng/ml PDGF-BB 48 h. The level of cell proliferation was detected by an EdU assay. Scale bar = 100 μm. **(B)** The effect of AdIRS-1 on the migration level of PASMCs induced by PDGF-BB by the wound healing assay. PASMCs were starved in a serum-free medium for 24 h after being infected with AdIRS-1 (MOI = 100) for 48 h. We scratched the bottom of the six-well plate with a 1 ml sterile pipette tip, then added PDGF-BB (20 ng/ml) to induce cells for 48 h. Images were taken at 0 h and 48 h. Scale bar = 250 μm. Data are expressed as mean ± standard deviation (mean ± SD), *n* = 5. *P < 0.05. Ctrl, control group; PDGF, 20 ng/ml PDGF-BB-induced PASMCs group; EV, empty vector; AdIRS-1, adenovirus expressing EGFP and IRS-1.

To understand the function of IRS-1 in the regulation of PASMC proliferation and migration, AdIRS-1 cells were generated and used to infect PASMCs. Overexpression of IRS-1 inhibited the proliferation (Figure 8A) and migration (Figure 8B) rates of PASMCs, as determined by EdU incorporation assay and wound healing assay, respectively. In a word, overexpression of IRS-1 inhibited the proliferation and migration rates of PASMCs stimulated by PDGF-BB.

## 4 Discussion

In this study, we have demonstrated that dysregulation of the PKC/IRS-1/ERK signaling pathway plays a critical role in pulmonary vascular remodeling in PAH by promoting the proliferation and migration of PASMCs. Our findings provide new insights into the molecular mechanisms underlying PAH and suggest potential therapeutic targets for intervention.

Our *in vivo* experiments using the MCT-induced PAH rat model revealed significant pathological changes of PAH development. We observed elevated mPAP and RVHI, indicating increased pulmonary artery pressure and right ventricular hypertrophy. Histological analysis showed thickening of the pulmonary arterial walls, with increased WT% and WA%, reflecting pulmonary vascular remodeling. These results confirm the successful establishment of the PAH model and highlight the structural alterations associated with the disease. At the molecular level, we found that the phosphorylation levels of PKC, IRS-1 (ser 318), and ERK were significantly increased in the lung tissues of PAH rats compared to controls, while the total protein level of IRS-1 was decreased. These changes suggest that activation of the PKC/IRS-1/ERK signaling pathway is associated with the pathological state of PAH. The increased PKC activity may lead to enhanced serine phosphorylation of IRS-1, promoting its degradation and resulting in decreased IRS-1 protein levels. This process potentially shifts signaling towards the ERK pathway, promoting PASMCs proliferation and migration and contributing to vascular remodeling.

In our study, MCT was used to build experimental animal models of PAH, and PASMCs stimulated by PDGF-BB were used as cellular models for further research (24). Research has demonstrated that growth factors, hormones, cytokines, and other factors can transiently activate MEK/ERK *in vitro* experiments, with peak activity typically occurring around 10 min after cellular interventions (26). However, the corresponding phenotypic changes in cells exhibit a time lag. In our *in vitro* experiments, we employed PDGF-BB to stimulate PASMCs. After a 15 min stimulation, a remarkable and rapid increase in the phosphorylation levels of PKC, IRS-1 (serine 318), and ERK was observed. Notably, the total protein levels of these components remained unaltered throughout this short-term stimulation, which clearly indicates the swift activation of the PKC/IRS-1/ERK signaling pathway. Subsequently, following a 48-hour PDGF-BB intervention, we detected a significant decrease in the total protein level of IRS-1. Concurrently, the proliferation and migration capabilities of PASMCs were markedly enhanced. These experimental observations not only strongly support the findings from our *in vivo* studies but also suggest that the protracted activation of this pathway triggers the degradation of IRS-1 and promotes the pathological behaviors of cells, thereby providing valuable insights into the underlying molecular mechanisms.

**Figure 9:**
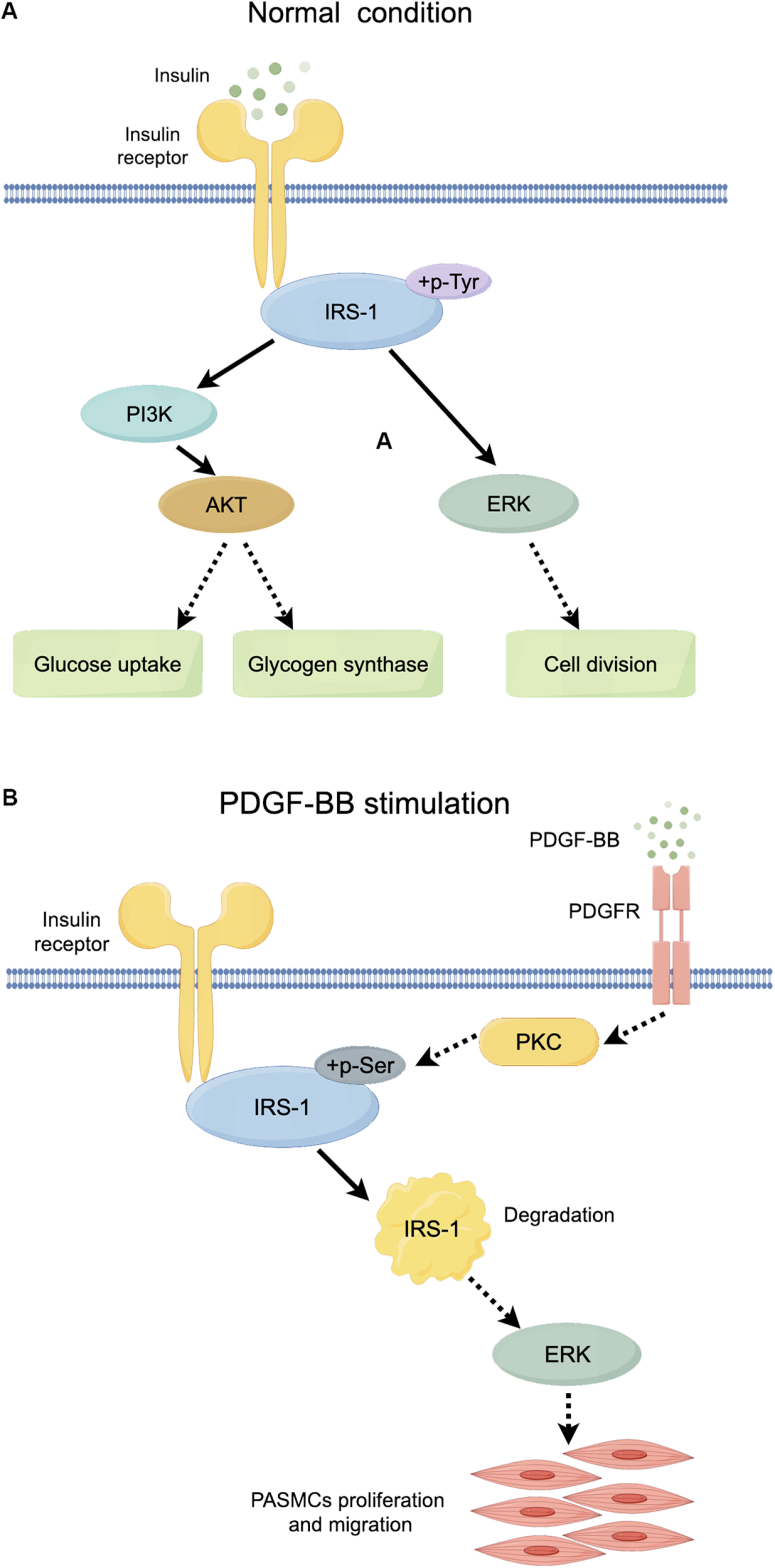
Schematic diagram of the role of IRS-1 under normal conditions and PDGF-BB stimulation. **(A)** Mechanistic map of insulin action through the PI3K and ERK signaling pathways. **(B)** Diagram of the role of PKC/IRS-1/ERK signaling pathway in PDGF-BB-induced PASMCs proliferation and migration. Full line arrow: direct interaction; dashed line arrow: indirect interaction.

AdIRS-1 reversed the effects of PDGF-BB, reducing ERK phosphorylation and inhibiting PASMCs proliferation and migration. This suggests that maintaining IRS-1 levels can counteract the hyperproliferative phenotype of PASMCs in PAH. IRS-1 is a critical mediator of insulin signaling, and its degradation disrupts the balance between metabolic and mitogenic pathways (11–13, 15, 27, 28). By restoring IRS-1 expression, we can potentially normalize insulin signaling and reduce pathological PASMCs activity. These results underscore the therapeutic potential of targeting IRS-1 to restore normal insulin signaling and inhibit pathological vascular remodeling (Figure 9).

The link between insulin resistance and IRS-1 function provides a mechanistic explanation for our findings. Insulin resistance is known to increase serine phosphorylation of IRS-1, leading to its functional impairment and degradation (11–13). The loss of IRS-1 disrupts normal insulin signaling, particularly the PI3K/Akt pathway, and enhances activation of the ERK pathway (11–13, 29). This shift promotes cell proliferation and migration, contributing to vascular remodeling in PAH. Our research findings indicate that similar mechanisms also exist in PASMCs, where increased serine phosphorylation of IRS-1 leads to its degradation and subsequent ERK activation. Notably, IRS-1 and IRS-2 have different roles in PAH. While IRS-2 has been implicated in pulmonary vascular remodeling, IRS-1 appears to have a more direct role in promoting PASMCs proliferation and migration (10). IRS-1 specifically mediates mitogenic signaling pathways, such as the ERK pathway, making it a potential therapeutic target. By focusing on IRS-1, our study provides a clearer understanding of the molecular events that drive PASMCs dysfunction in PAH.

There is a large body of previous literature suggesting that under physiological conditions, binding of insulin to the insulin substrate receptor increases the level of tyrosine phosphorylation of IRS-1, activating the two cascades, PI3K and ERK (Figure 9A). However, our study mainly focuses on the serine phosphorylation of IRS-1 in the insulin-resistant state. PKC serves as an upstream regulator of IRS-1 in this signaling cascade. As a serine/threonine kinase (30), PKC can phosphorylate IRS-1 on serine residues, affecting its stability and signaling function (31, 32). An increase in PKC activity was observed in the vascular endothelial cells of patients with PAH, and elevated PKC activity has been detected in a rat model of hypoxia-induced pulmonary hypertension (17). At the same time, PKC activity was reported to be involved in the state of IR (33, 34). Our findings show that PKC activation leads to increased serine phosphorylation of IRS-1 (ser 318), promoting its degradation. Inhibition of PKC with Gö 6983 reduced IRS-1 serine phosphorylation, restored IRS-1 protein levels, decreased ERK phosphorylation, and inhibited PASMCs proliferation and migration. This suggests that targeting PKC could prevent IRS-1 degradation and normalize insulin signaling in PASMCs. Although p-ERK at 48 h no longer exceeds the control level, the early (15 min) surge in ERK phosphorylation is key to initiating the pro-proliferative and pro-migratory events. Overexpression of IRS-1 can reduce the baseline level of ERK phosphorylation (Figure 7D), much like the inhibitory impact exerted by Gö 6983 pretreatment on the baseline level of ERK phosphorylation (Figure 5C). This reduction in the baseline level subsequently suppresses the early peak level of ERK phosphorylation. Therefore, the net “pro-growth” cascade occurring in the following several hours is diminished. In other words, even though ERK phosphorylation may appear to normalize by 48 h, the short-term peak is critical in driving cellular outcomes. By controlling that early signal, IRS-1 overexpression effectively reduces the downstream proliferation and migration measured at later time points. Other kinases, such as inhibitor of kappa B kinase beta (IKKβ) and glycogen synthase kinase 3 (GSK-3) (29, 35, 36), are also associated with IRS-1 serine phosphorylation. Inflammatory factors like tumor necrosis factor-alpha (TNF-α) can activate IKKβ, leading to IRS-1 serine phosphorylation and insulin resistance (37). These factors may contribute to IRS-1 dysregulation in PASMCs, and their roles warrant further investigation. Understanding how these upstream activators influence IRS-1 function could reveal additional targets for therapeutic intervention.

The implications for therapeutic strategies are significant. Current PAH treatments mainly focus on vasodilation and reducing pulmonary vascular resistance but do not directly address the underlying cellular proliferation and migration. Targeting the PKC/IRS-1/ERK pathway offers a novel approach that could inhibit pulmonary vascular remodeling. Potential strategies include the use of PKC inhibitors like Gö 6983 and methods to enhance IRS-1 expression or stability. By preventing IRS-1 degradation and normalizing insulin signaling, these interventions could reduce PASMCs proliferation and migration, slowing or reversing vascular remodeling. In addition, Gö 6983 was demonstrated to suppress the proliferation of rat goblet cells stimulated by epidermal growth factor (EGF) (23). PDGF-BB binding to the PDGF receptor was observed to affect the activity of PKC (35).

Our study has limitations. We did not validate our findings using clinical samples from PAH patients, so human studies are necessary to confirm the applicability of our results. Additionally, we did not perform *in vivo* therapeutic interventions using PKC inhibitors or IRS-1 modulators; evaluating these interventions in animal models is an important next step.

In summary, our study demonstrates that dysregulation of the PKC/IRS-1/ERK signaling pathway contributes to pulmonary vascular remodeling in PAH by promoting PASMCs proliferation and migration. Targeting this pathway offers new therapeutic possibilities beyond current vasodilator therapies. By inhibiting PKC activity or enhancing IRS-1 stability, it may be possible to prevent or reverse the pathological changes in PAH.

## Data Availability

The original contributions presented in the study are included in the article/Supplementary Material, further inquiries can be directed to the corresponding author/s.
