# Supplementary material for: Role of insulin signaling dysregulation in pulmonary vascular remodeling in rats with monocrotaline-induced pulmonary arterial hypertension
Source: Front Cardiovasc Med. 2025 Mar 24;12:1543319. doi: 10.3389/fcvm.2025.1543319 (PMC11973325; doi:10.3389/fcvm.2025.1543319)
Supplement: Supplementary file 1 [file Datasheet1.pdf]

fig2

original blots in supplementary information

Cropped blots in manuscript

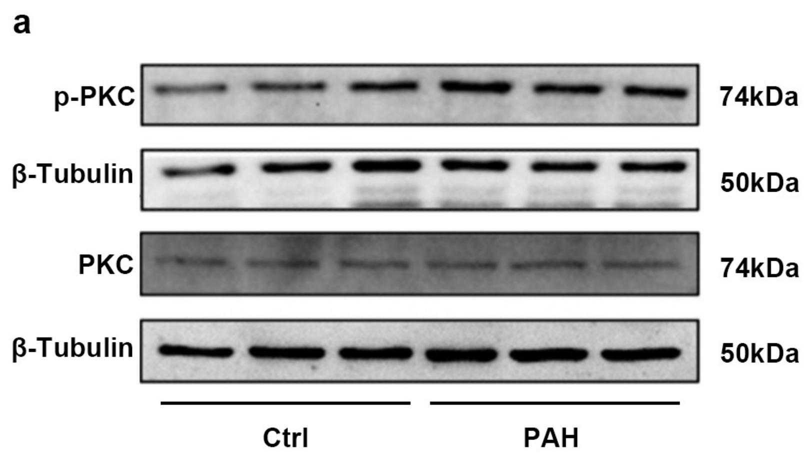

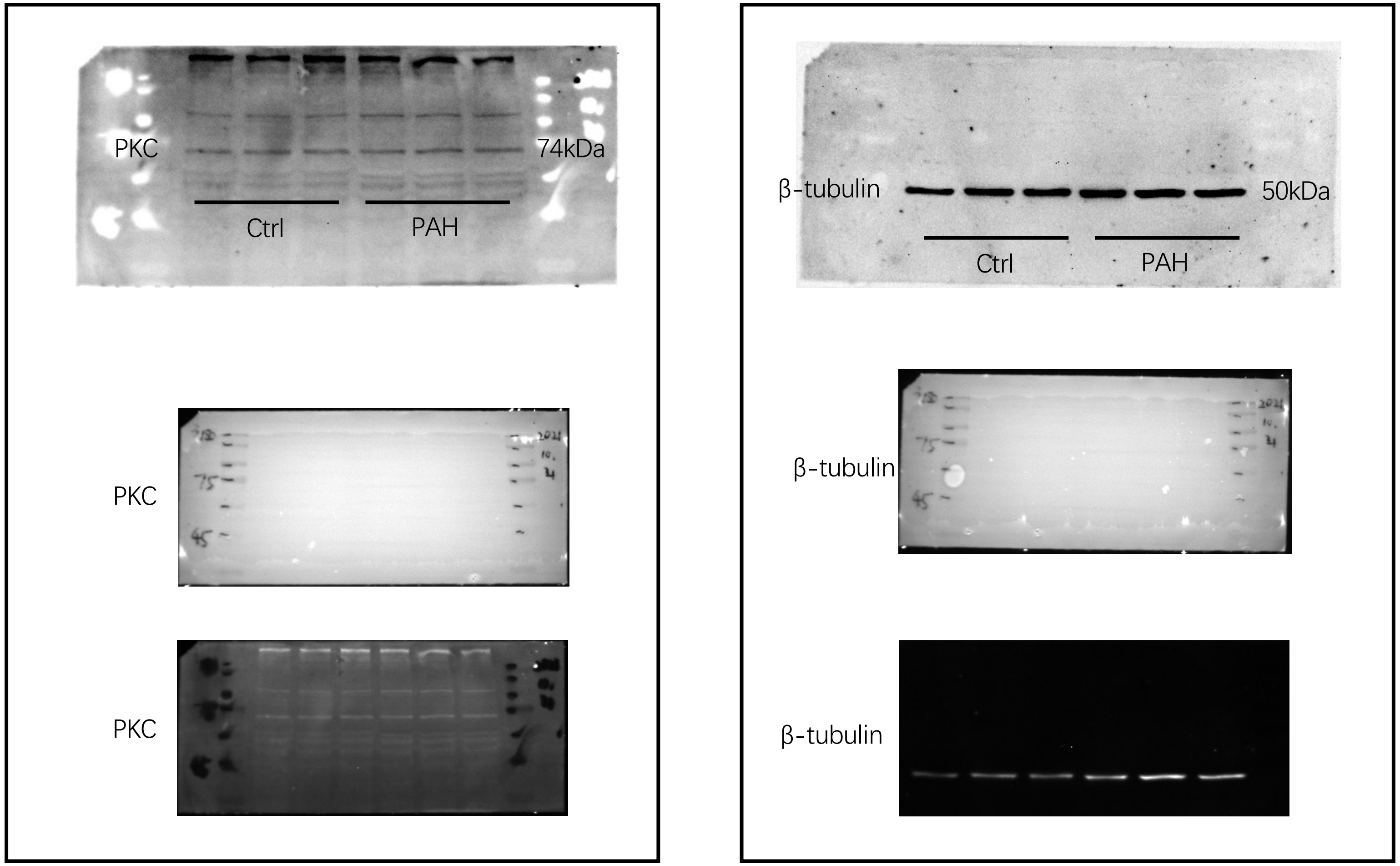

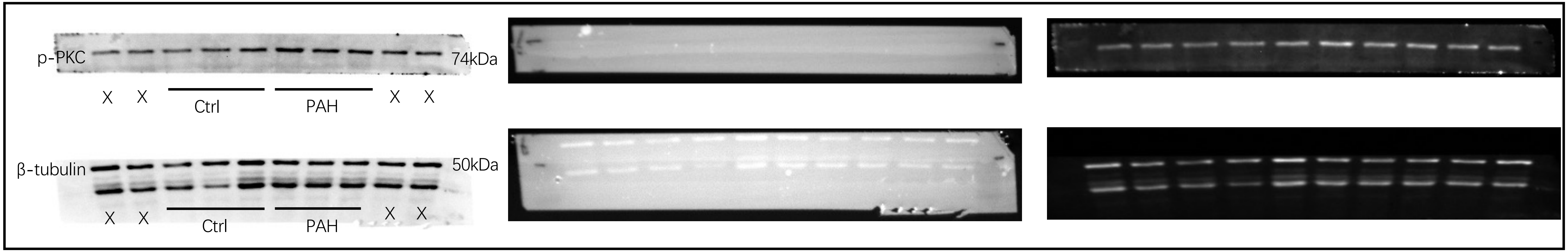

fig2

b

Cropped blots in manuscript

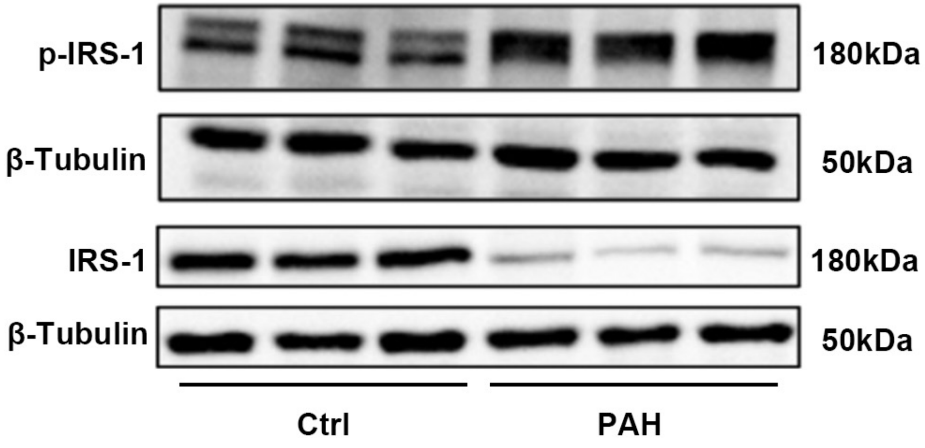

original blots in supplementary information

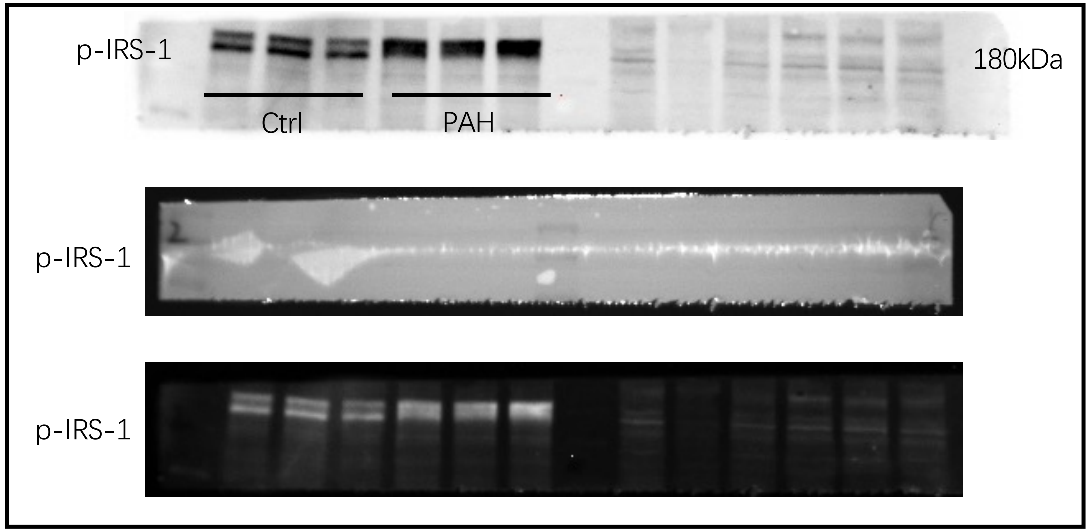

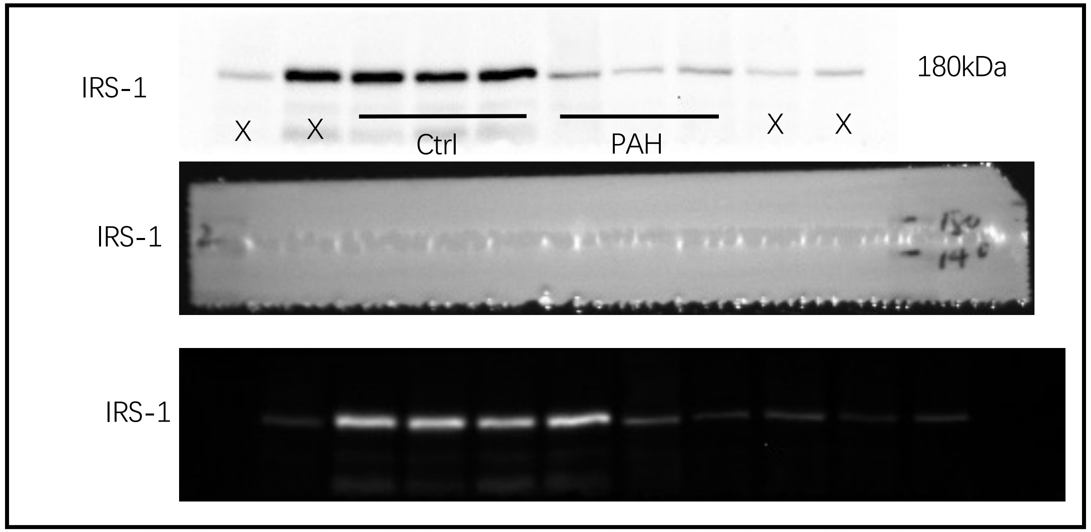

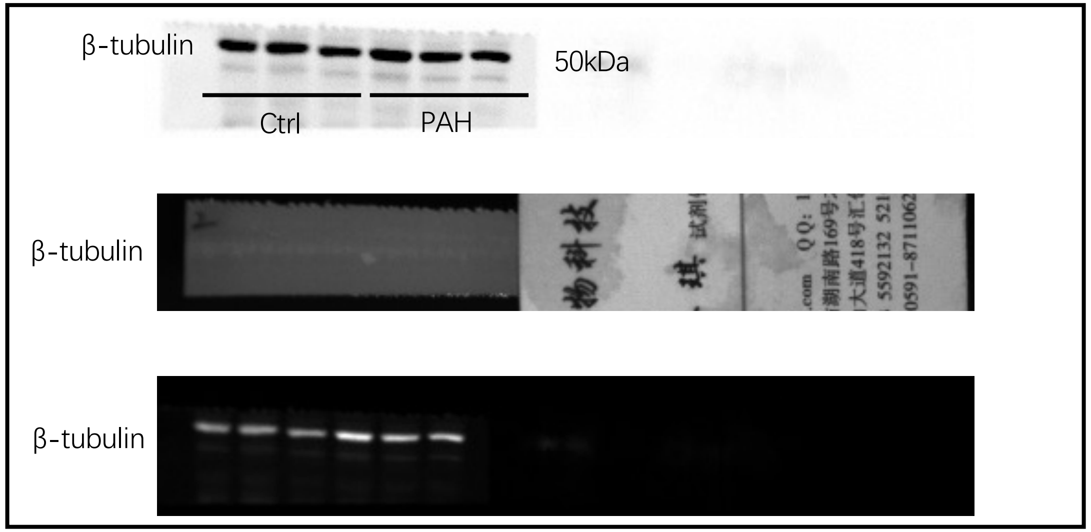

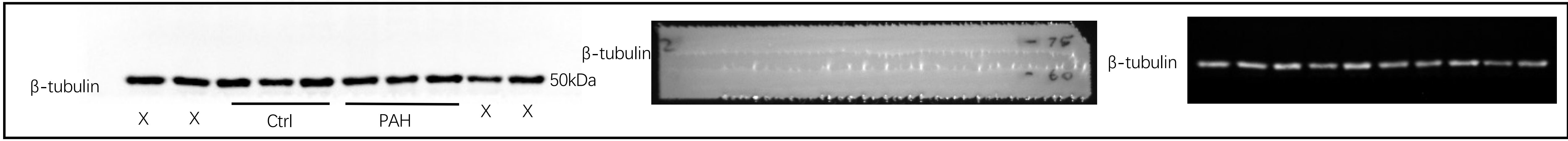

fig2

original blots in supplementary information

Cropped blots in manuscript

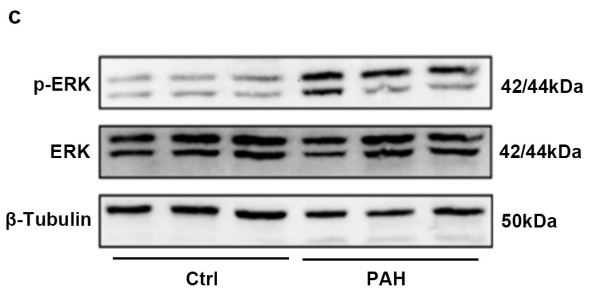

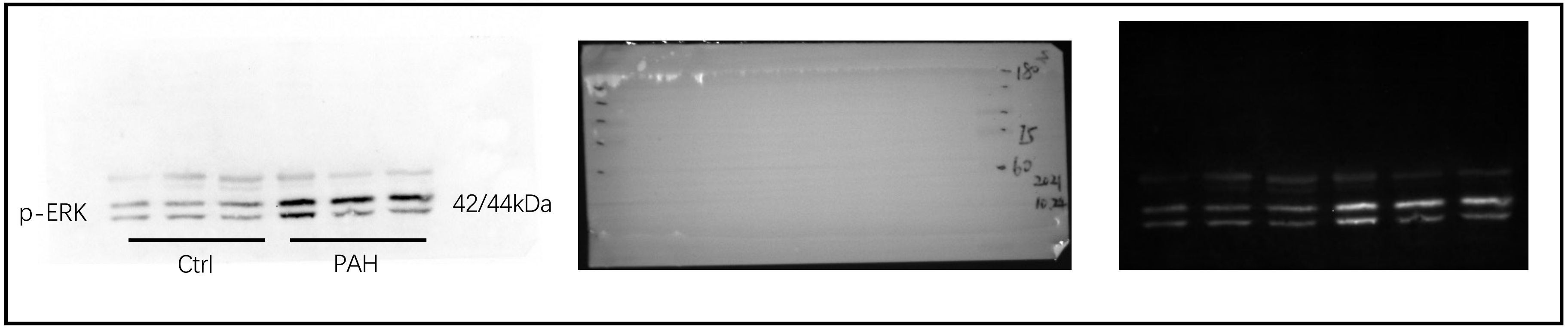

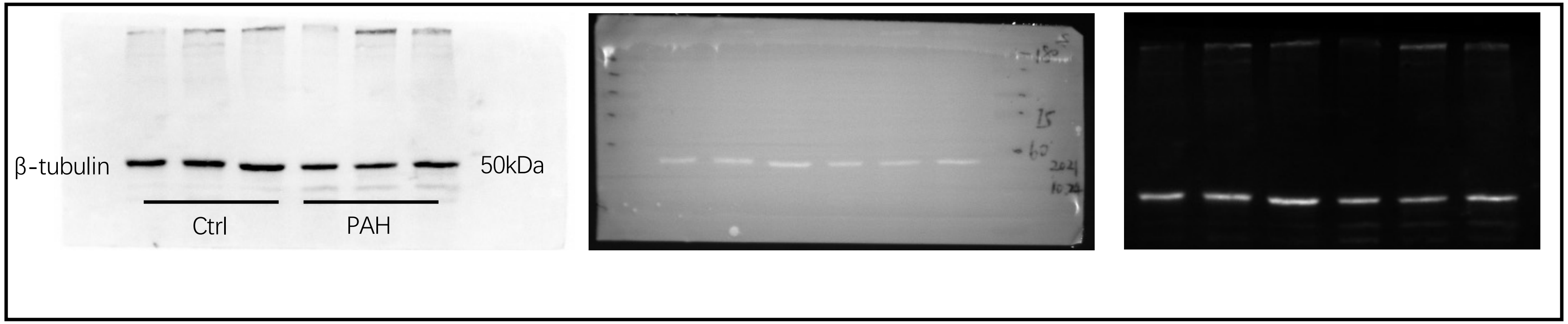

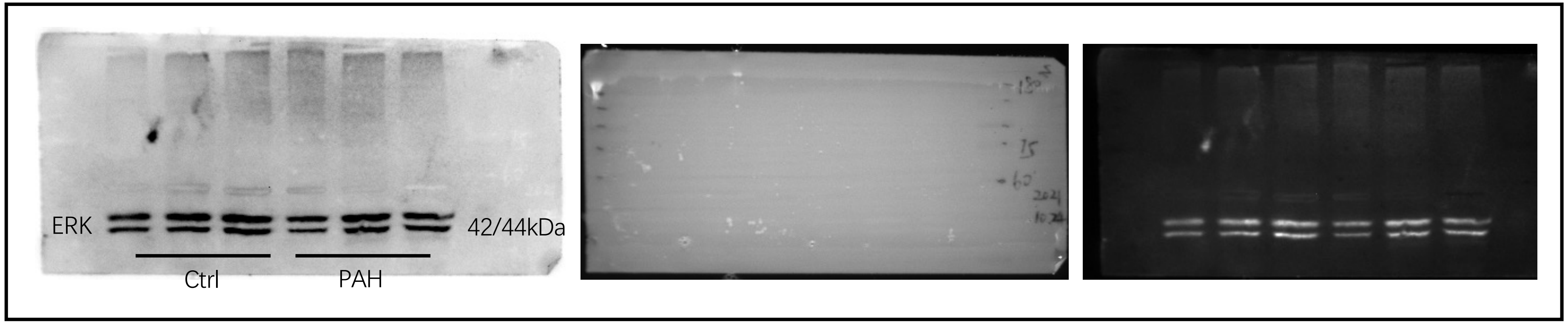

fig4

original blots in supplementary information

Cropped blots in manuscript

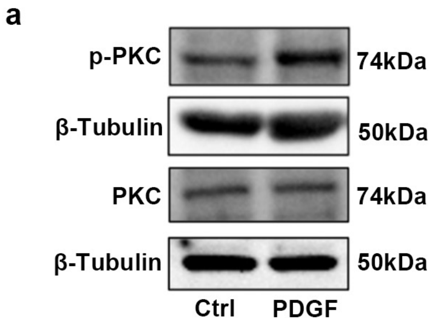

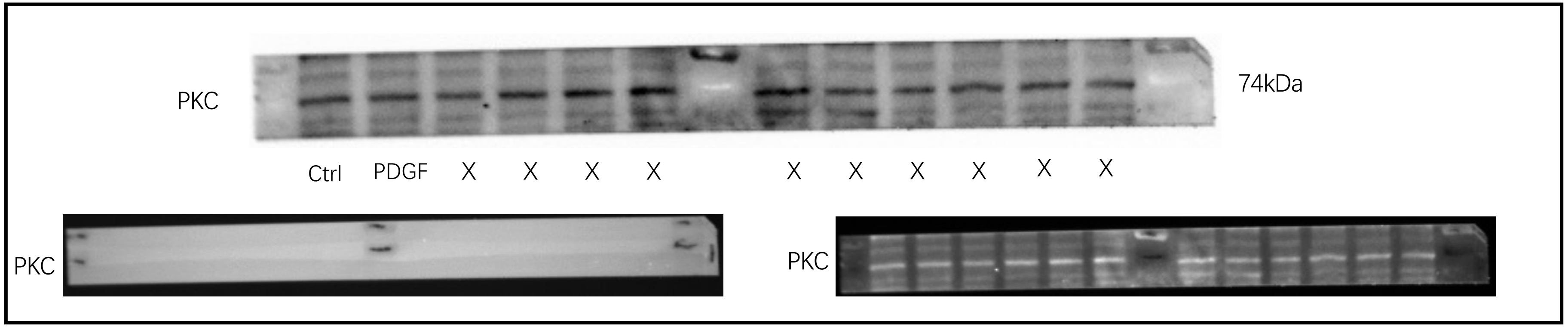

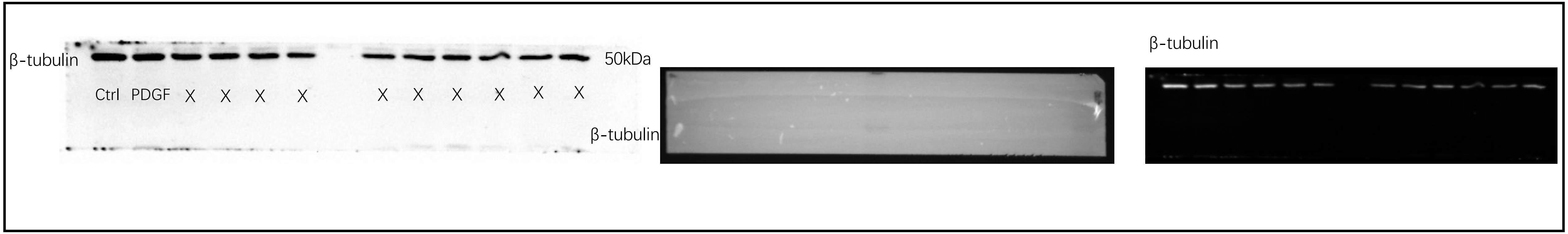

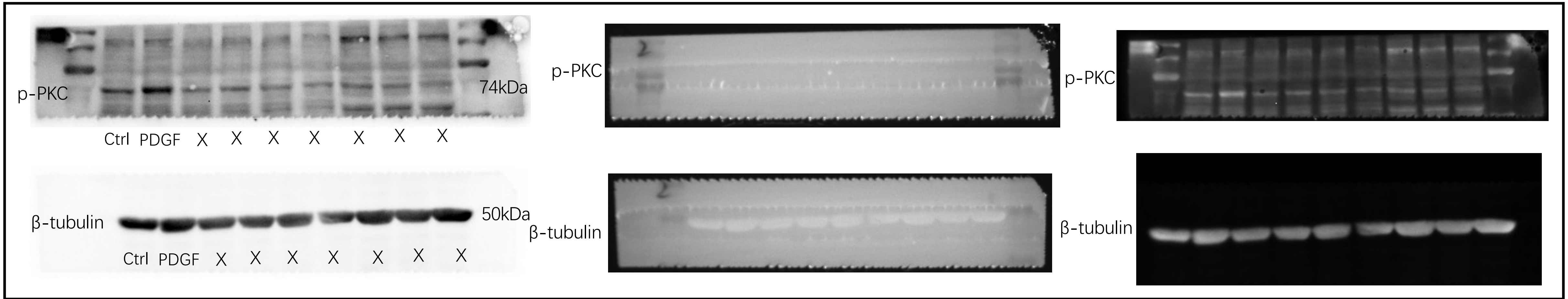

fig4

original blots in supplementary information

Cropped blots in manuscript

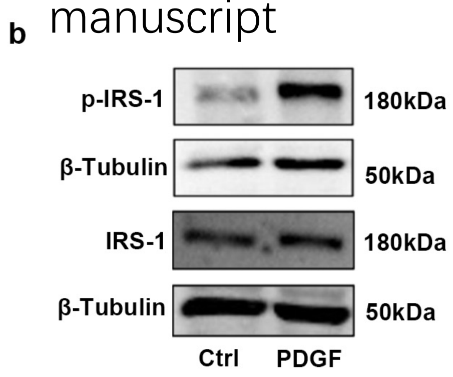

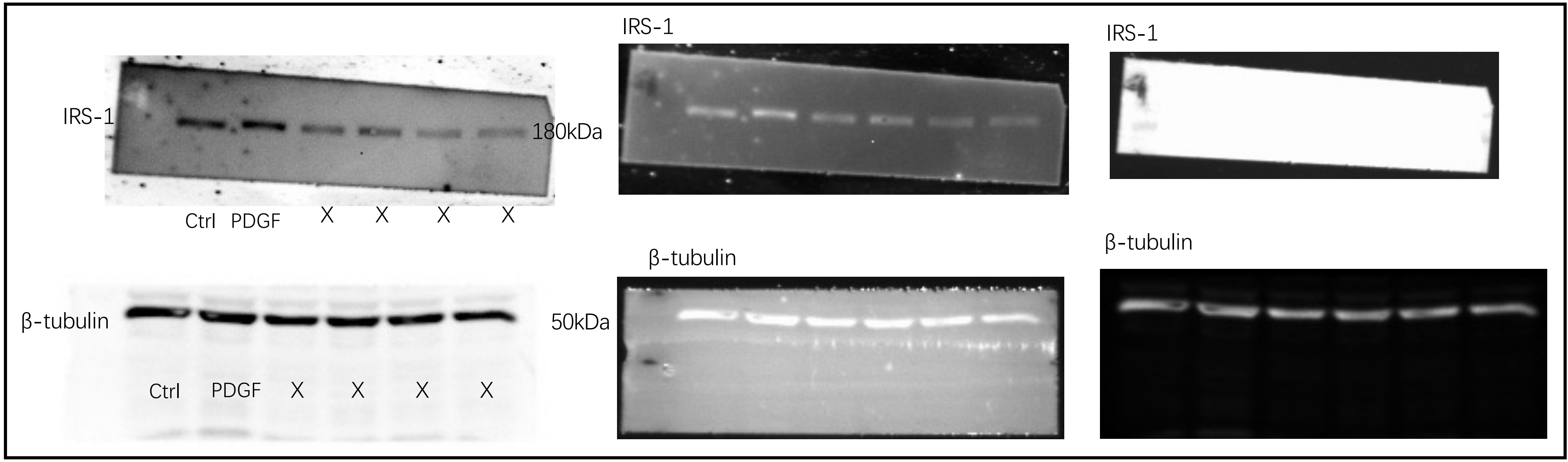

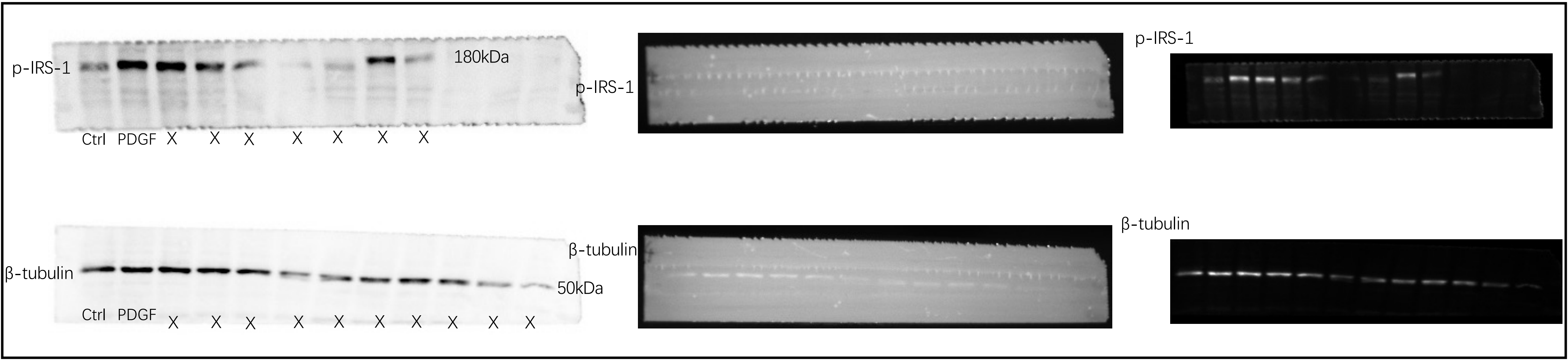

fig4

c Cropped blots in manuscript

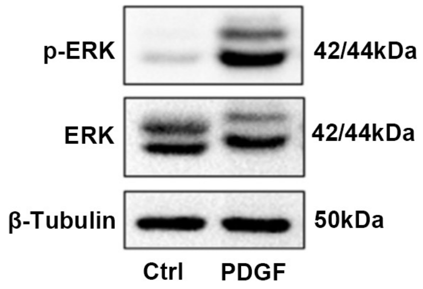

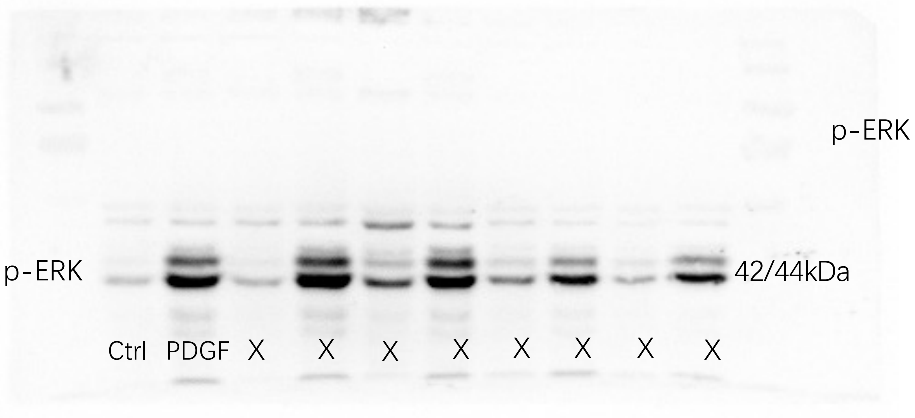

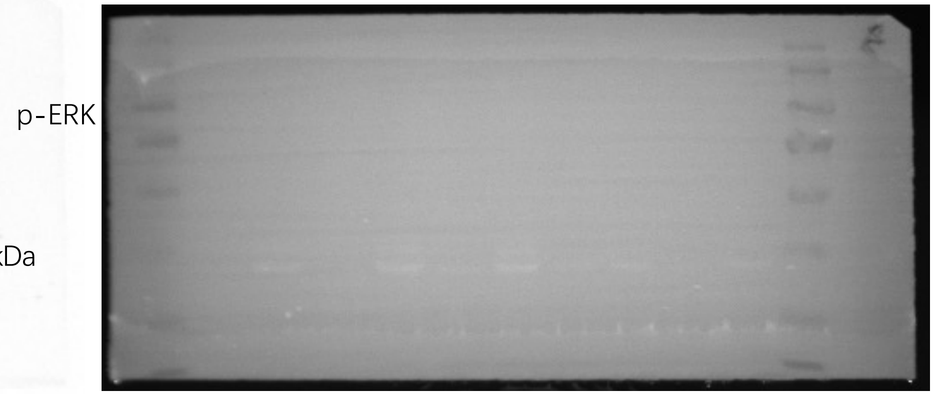

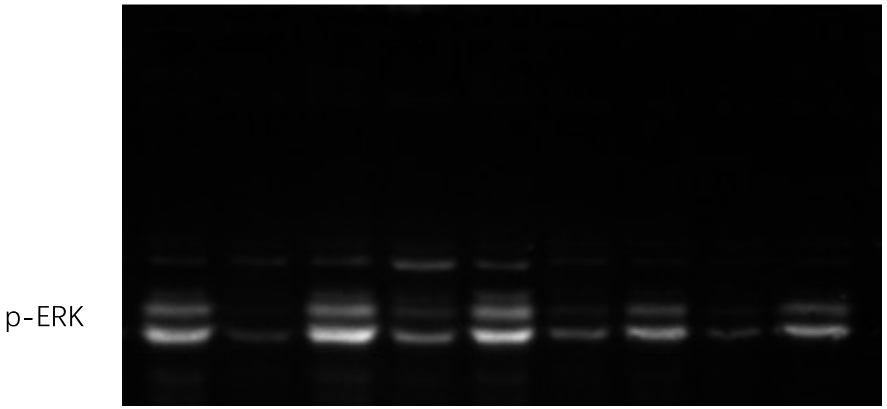

original blots in supplementary information

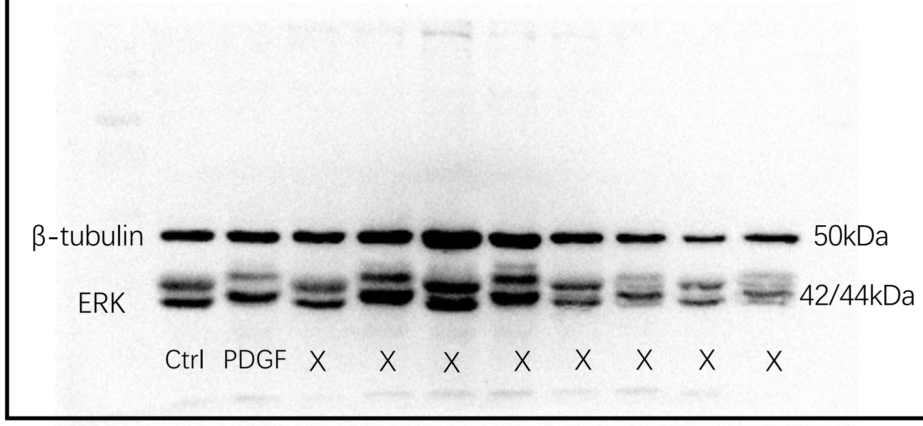

original blots in supplementary information

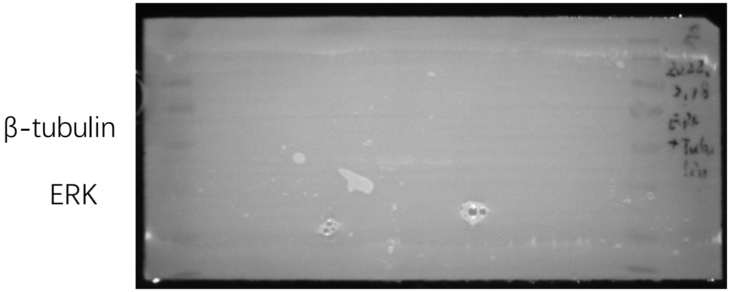

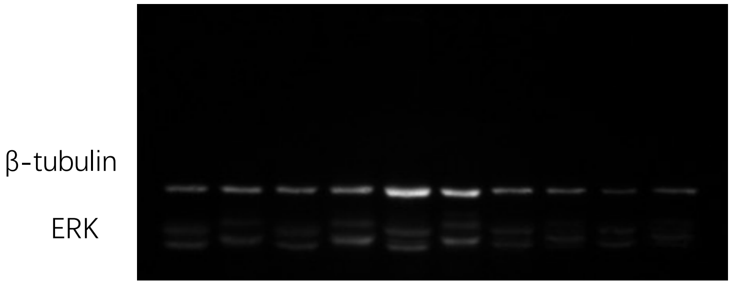

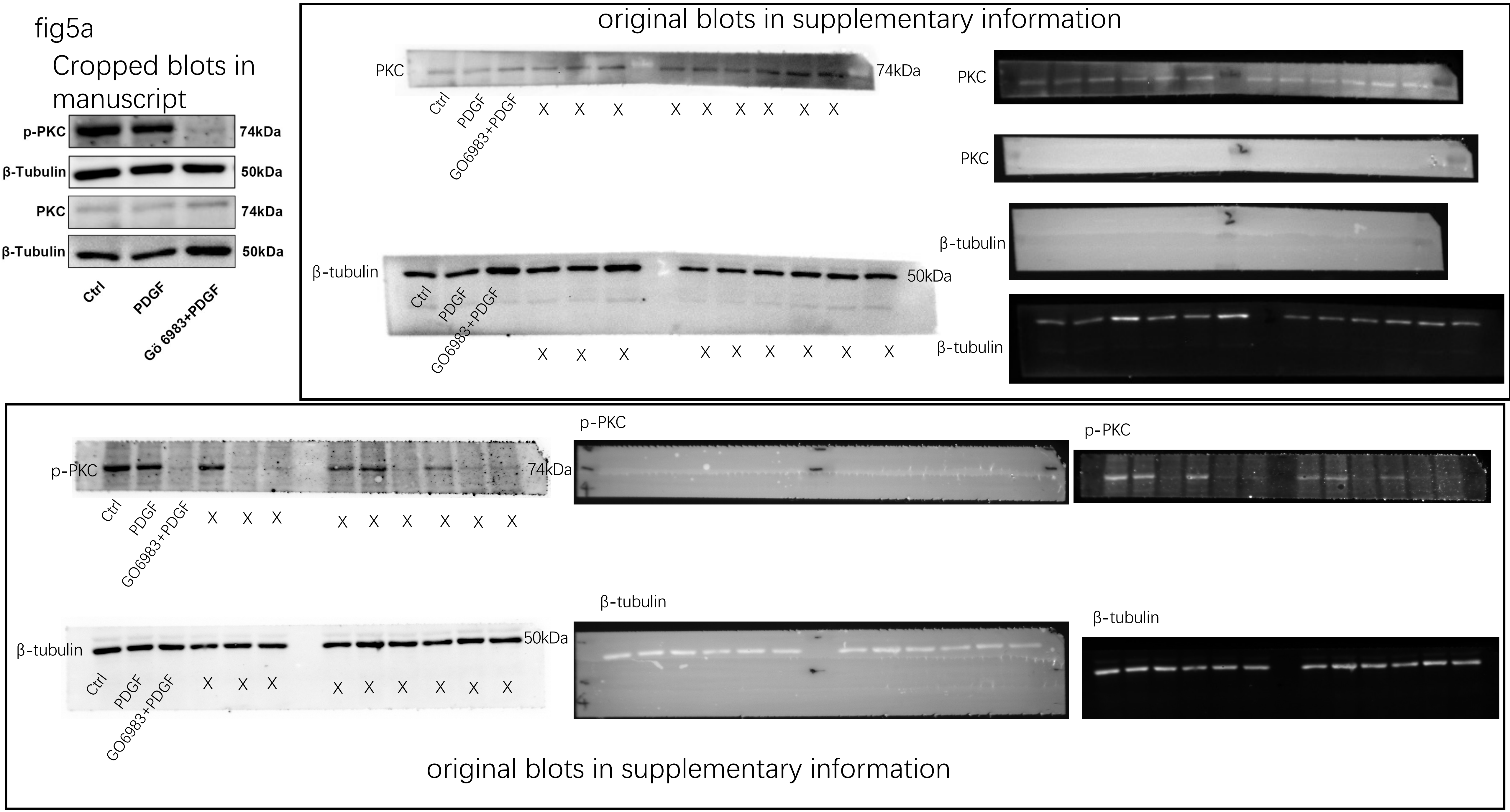
fig5a
Cropped blots in manuscript
p-PKC
74kDa
β-Tubulin
50kDa
PKC
74kDa
β-Tubulin
50kDa
Ctrl
PDGF
Gö 6983+PDGF
original blots in supplementary information
PKC
74kDa
Ctrl
PDGF
GO6983+PDGF
X X X X X X X X X
PKC
PKC
β-tubulin
β-tubulin
50kDa
Ctrl
PDGF
GO6983+PDGF
X X X X X X X X X
β-tubulin
p-PKC
74kDa
Ctrl
PDGF
GO6983+PDGF
X X X X X X X X X
p-PKC
p-PKC
β-tubulin
50kDa
Ctrl
PDGF
GO6983+PDGF
X X X X X X X X X
β-tubulin
β-tubulin
original blots in supplementary information

fig5b

Cropped blots in manuscript

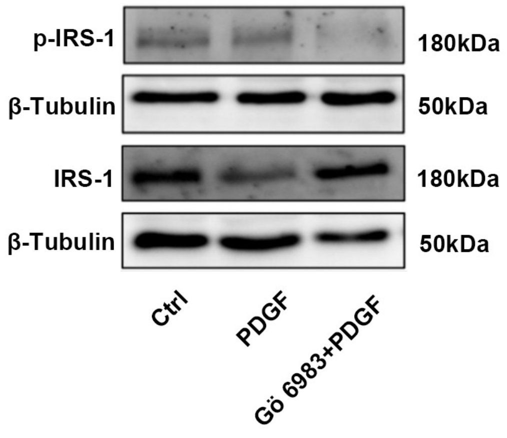

original blots in supplementary information

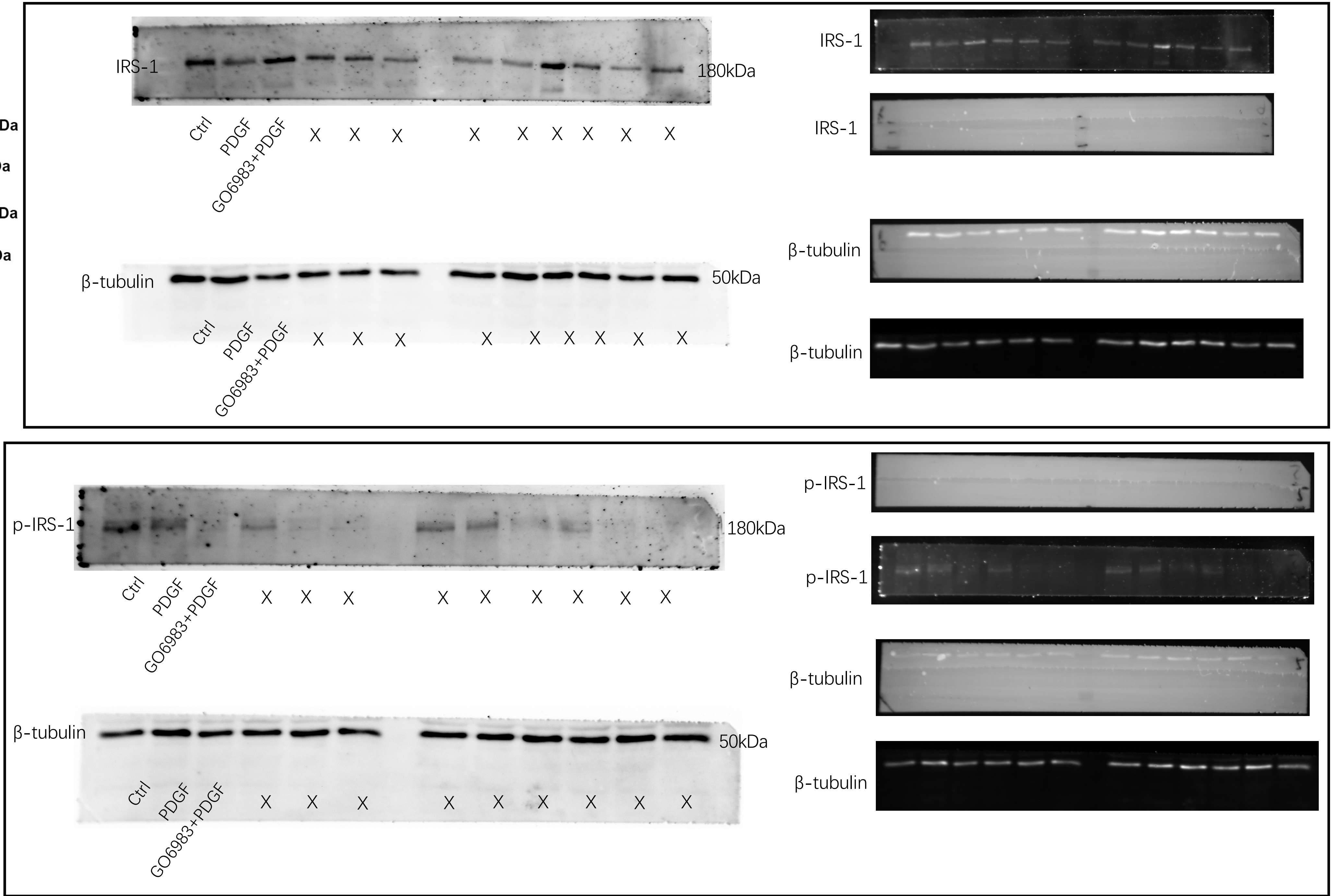

fig5

Cropped blots in manuscript

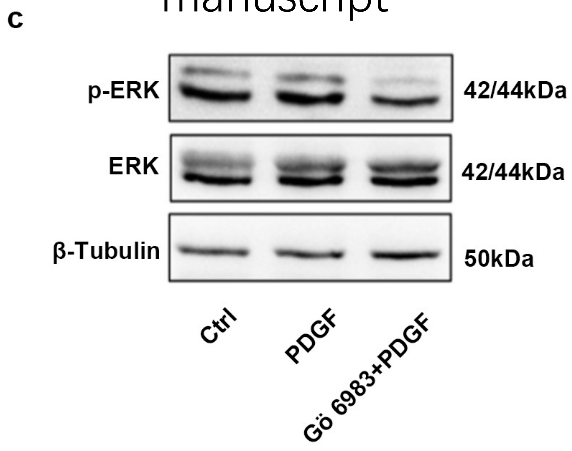

original blots in supplementary information

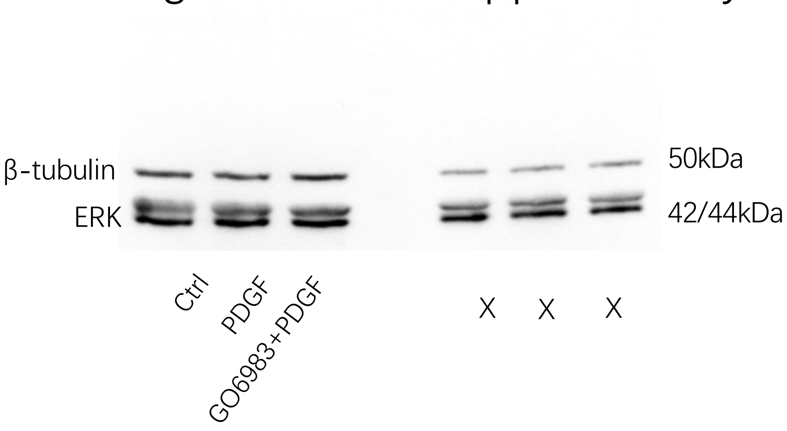

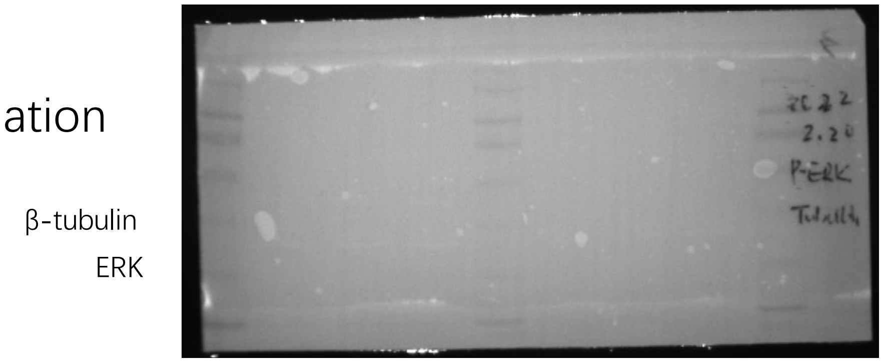

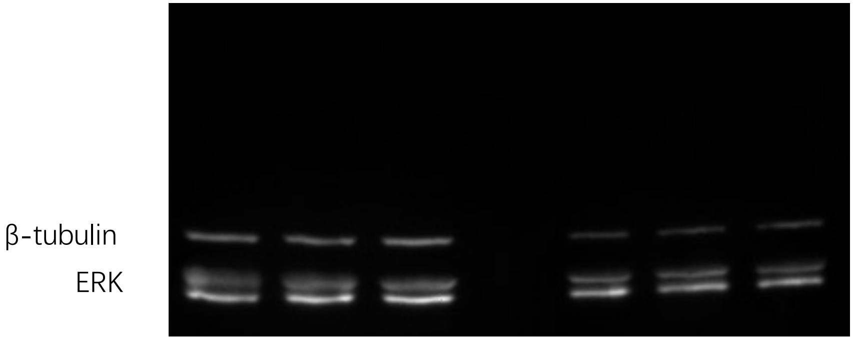

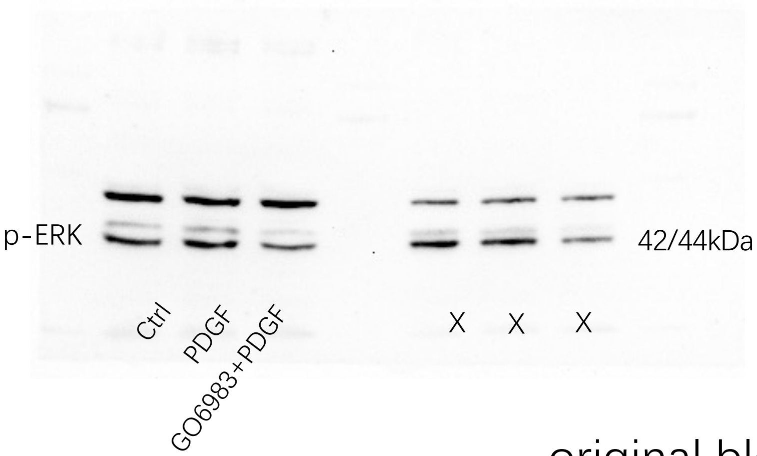

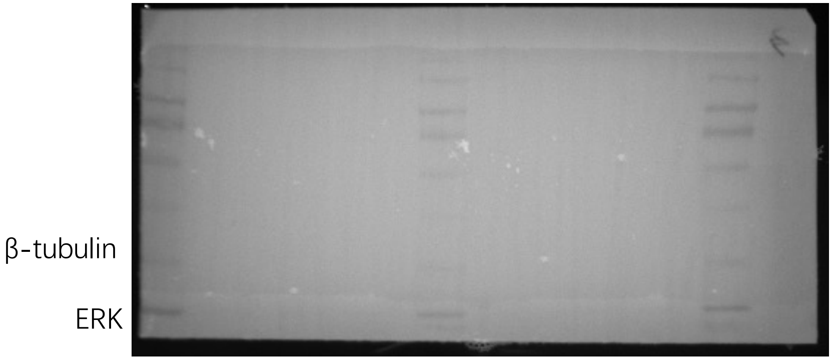

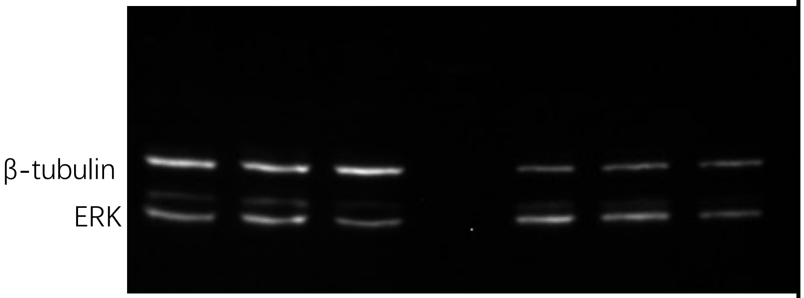

original blots in supplementary information

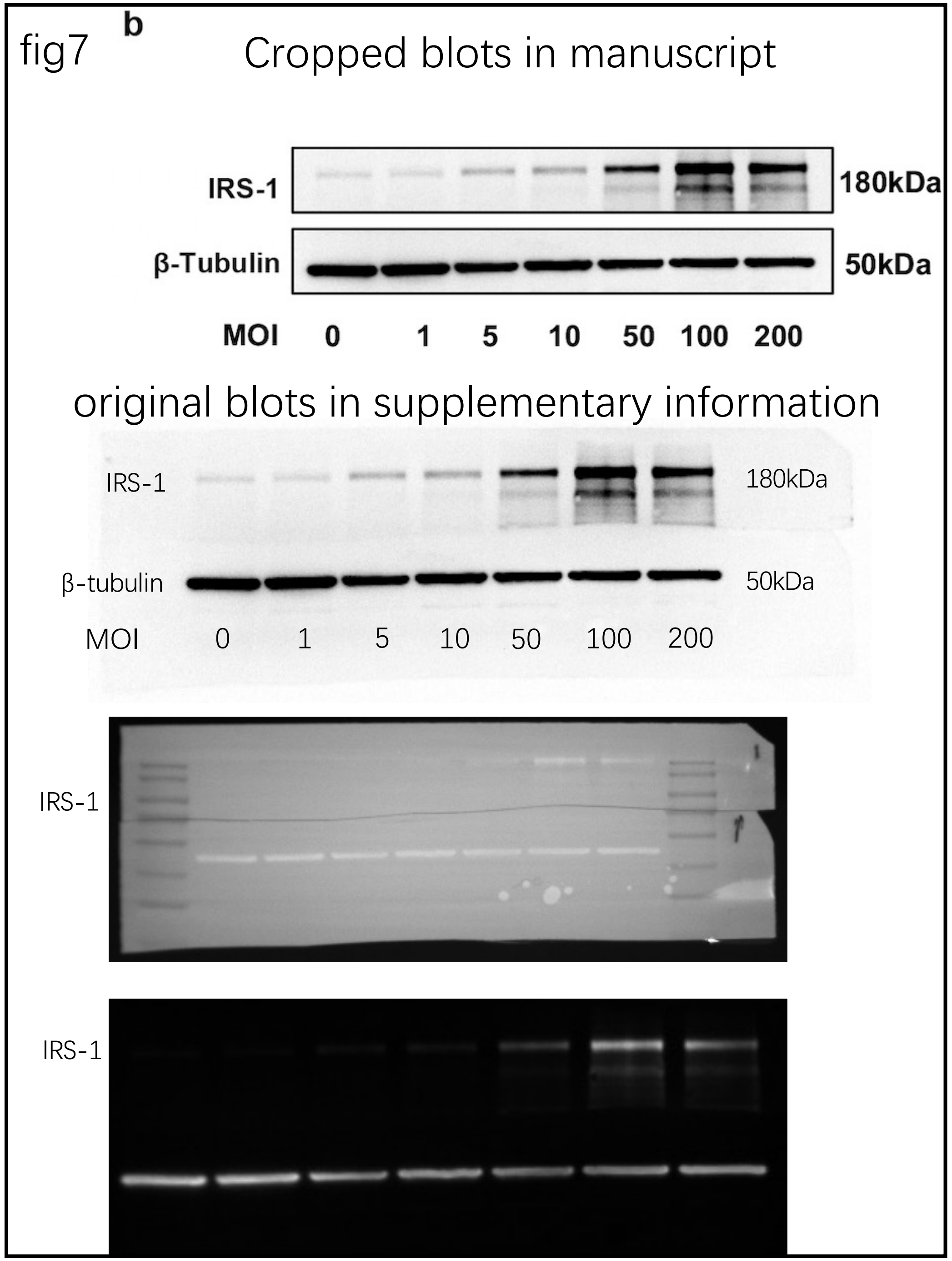
fig7
b
Cropped blots in manuscript
IRS-1
180kDa
β-Tubulin
50kDa
MOI 0 1 5 10 50 100 200
original blots in supplementary information
IRS-1
180kDa
β-tubulin
50kDa
MOI 0 1 5 10 50 100 200
IRS-1
IRS-1

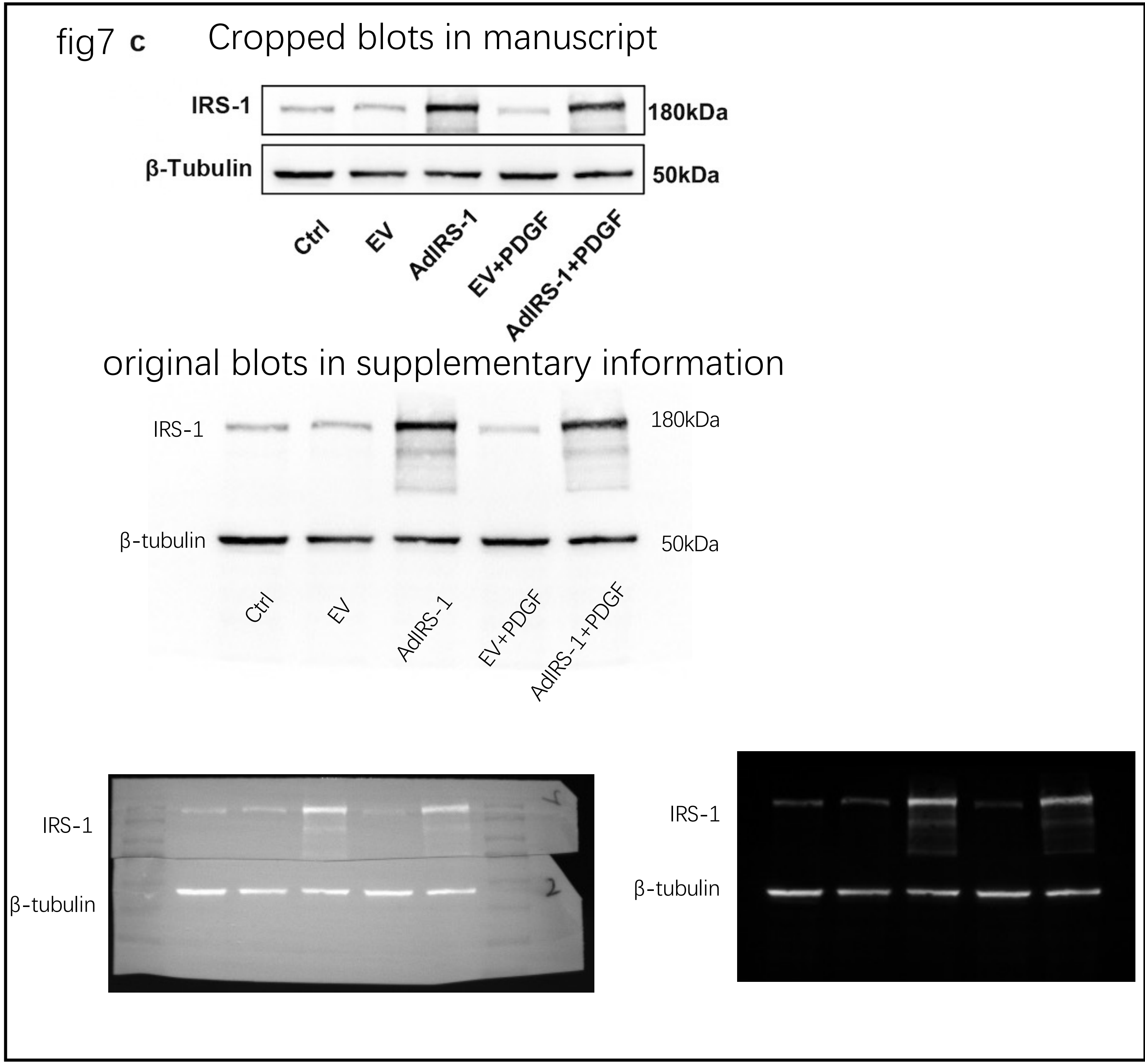
fig7 c
Cropped blots in manuscript
IRS-1
180kDa
β-Tubulin
50kDa
Ctrl
EV
AdIRS-1
EV+PDGF
AdIRS-1+PDGF
original blots in supplementary information
IRS-1
180kDa
β-tubulin
50kDa
Ctrl
EV
AdIRS-1
EV+PDGF
AdIRS-1+PDGF
IRS-1
β-tubulin
IRS-1
β-tubulin

fig7

Cropped blots in manuscript

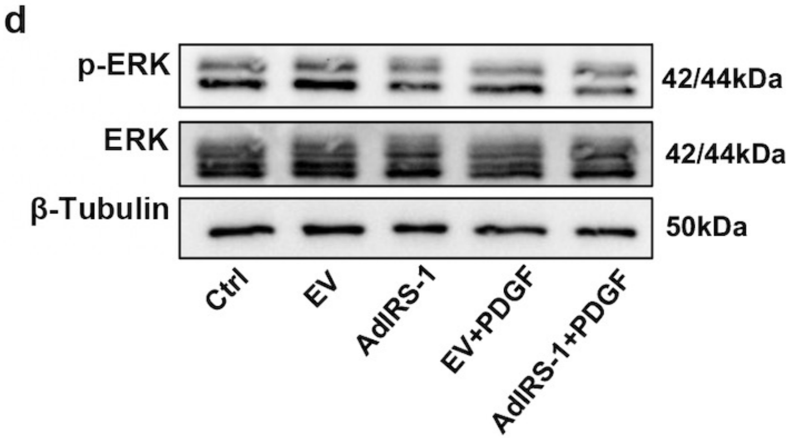

original blots in supplementary information

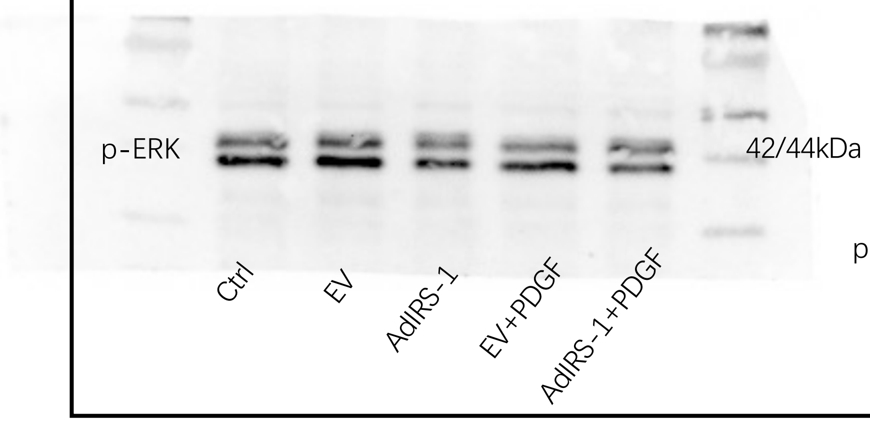

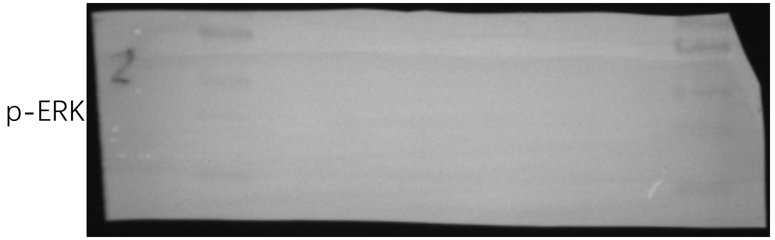

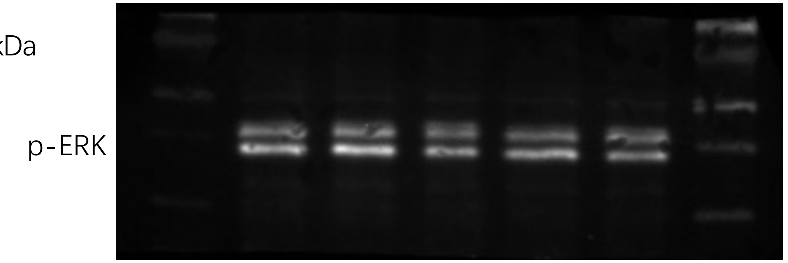

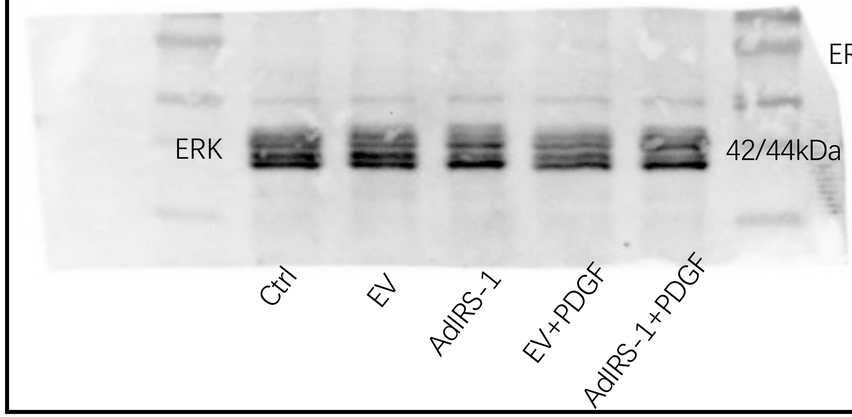

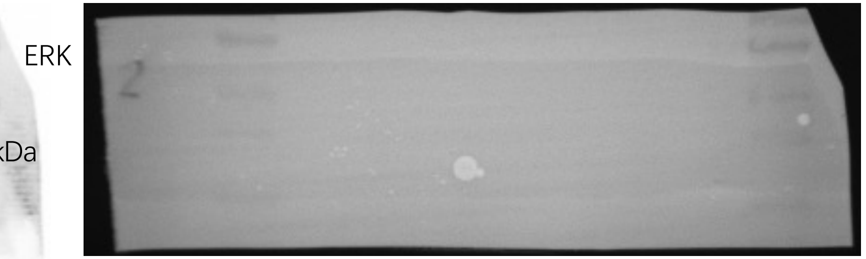

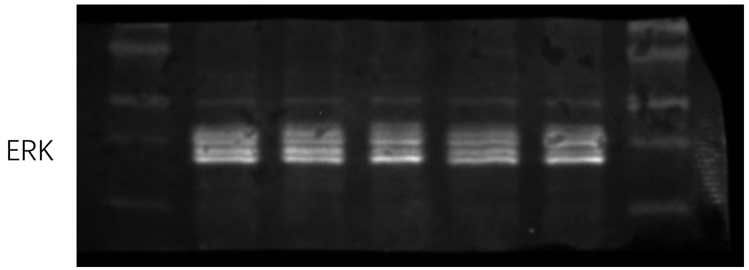

original blots in supplementary information

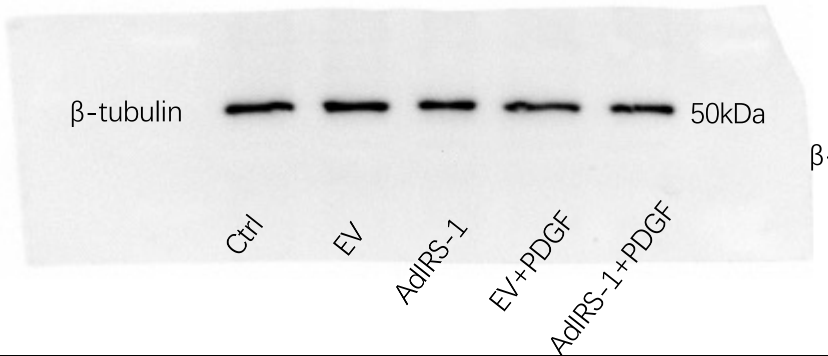

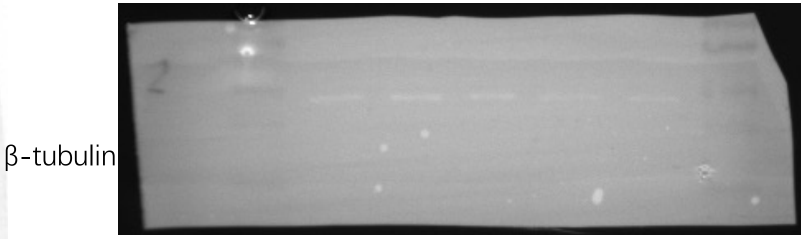

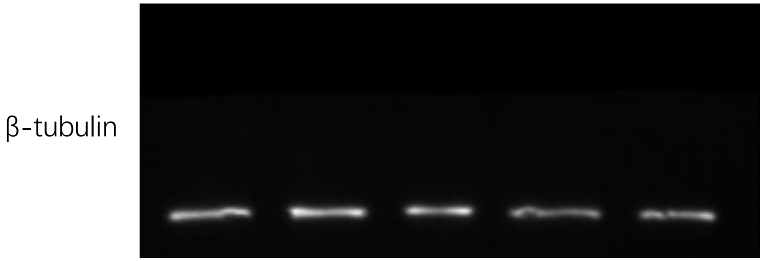

original blots in supplementary information
